# Audit lead selection and yield prediction from historical tax data using artificial neural networks

**DOI:** 10.1371/journal.pone.0278121

**Published:** 2022-11-30

**Authors:** Trevor Chan, Cheng-En Tan, Ilias Tagkopoulos

**Affiliations:** 1 Department of Computer Science, University of California, Davis, California, United States of America; 2 Genome Center, University of California, Davis, California, United States of America; Hanyang University, REPUBLIC OF KOREA

## Abstract

Tax audits are a crucial process adopted in all tax departments to ensure tax compliance and fairness. Traditionally, tax audit leads have been selected based on empirical rules and randomization methods, which are not adaptive, may miss major cases and can introduce bias. Here, we present an audit lead tool based on artificial neural networks that have been trained and evaluated on an integrated dataset of 93,413 unique tax records from 8,647 restaurant businesses over 10 years in the Northern California, provided by the California Department of Tax and Fee Administration (CDTFA). The tool achieved a 40.1% precision and 58.7% recall (F1-score of 0.42) on classifying positive audit leads, and the corresponding regressor provided estimated audit gains (MAE of $155,490). Finally, we evaluated the statistical significance of various empirical rules for use in lead selection, with two out of five being supported by the data. This work demonstrates how data can be leveraged for creating evidence-based models of audit selection and validating empirical hypotheses, resulting in higher audit yields and more fair audit selection processes.

## 1. Introduction

Tax audits are an important part of tax collection as the main process to correct errors, increase compliance, and ensure a fair taxation system [1]. The process usually starts with candidate business or taxpayer selection through different criteria, then an experienced auditor evaluates the selection criteria to decide which returns to audit. A critical part of the process is the criteria and process by which the tax agencies select the audit candidates so that the utility of the audit process is maximized in terms of fairness, time spent, and outcome. Challenges in audit selection include the lack of universally accepted criteria of an optimal audit selection process, the complexity of the tax system and latent variables that make it hard to generalize, and the incompatibility of data that are usually not ready for direct application of machine learning methods. In addition, the audit selection process needs to protect privacy, reduce bureaucracy, ensure fairness, and avoid any bias, while maximizing resource utilization and benefits for the taxpayer and state [1].

**Fig 1:**
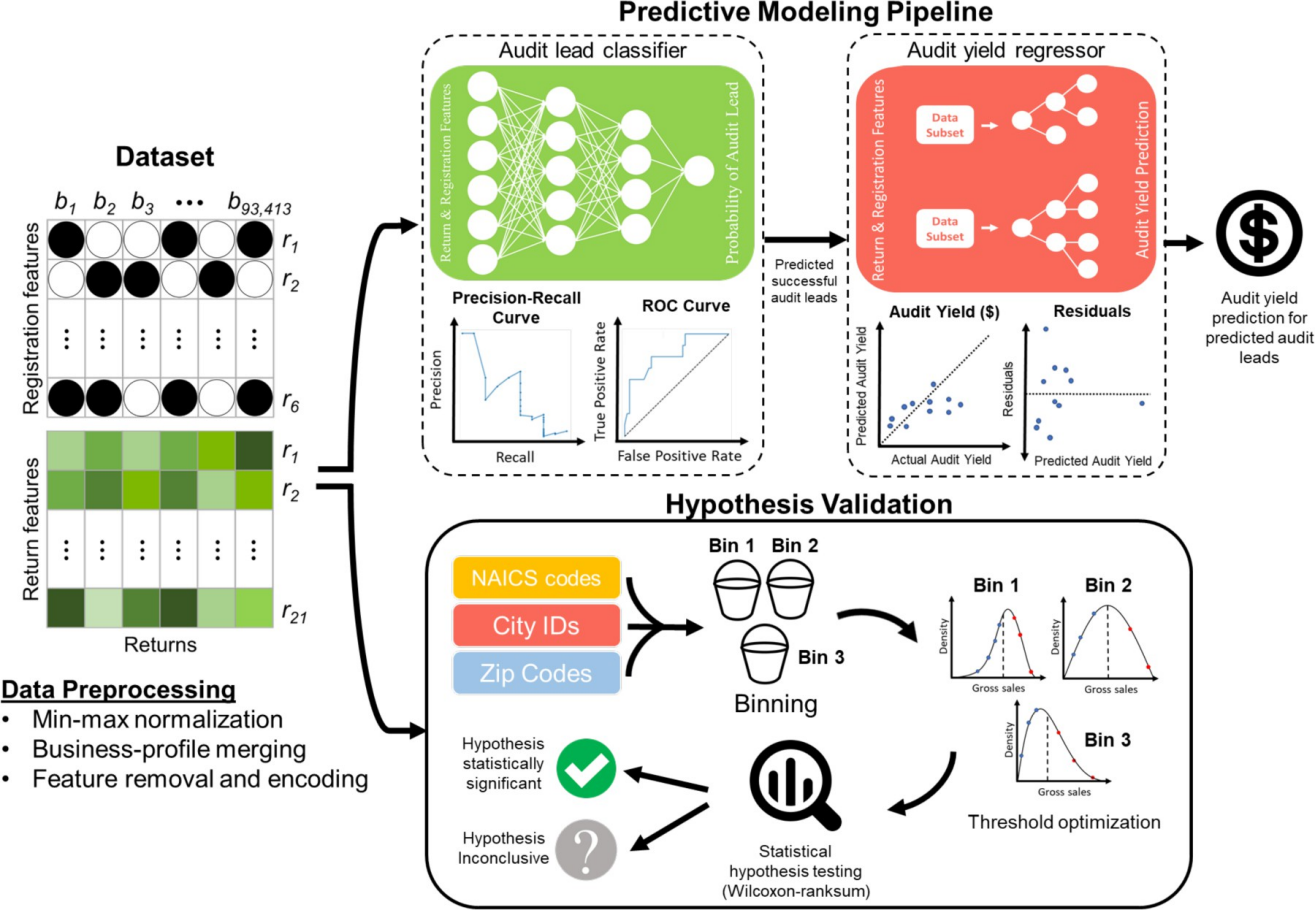
Overview of tax prediction pipeline–raw data received from the CDTFA is first preprocessed via normalization, merging of business profiles and feature removal to create a compendium of tax audit data based on registration and return features. This complete dataset then undergoes one of two steps: One, data is fed through a predictive modeling pipeline in which a feed-forward neural network first classifies an audit as positive or negative, and then second a series of extreme gradient boosted trees are used to predict audit yield based on these positive audits. Two, data is binned based on city and NAICS codes such that hypotheses generated by the CDTFA team can be tested and validated statistically.

For these reasons, agencies such as the IRS use computational tools to assist in their audit selection process. Most of those tools use different scoring systems [2] to identify discrepancies or rank reports based on the expected numbers. For instance, discriminant function (DIF) systems statistically determine the accuracy of a tax return over several hundred variables [3], while systems that use unreported income discriminant function (UIDIF) score returns based on unreported income potential, which is decided by examining expense and income ratios [4]. Similarly, systems use information returns processing (IRP) modules to cross-reference third-party data e.g. taxpayer income, employment, etc. with reported auditee information [5]. An auditor would usually take these reports into account, together with contextual and complementary sources of information, such as related activities, investment transactions, and social security information to form a recommendation on whether an audit would be warranted. This process creates a structured approach for audit selection, with some predefined criteria and quantifiable evidence. At the same time, auditor intuition is still the determining factor. Given current computational methods are limited in their scope, use simple computational methods that underfit in terms of model complexity, are usually based on empirical rules that have not been statistically validated for their accuracy, and do not learn from historical data and audit outcomes [6]. In collaboration with the California Department of Tax and Fee Administration (CDTFA), we integrated 93,413 unique tax records from 8,647 businesses over 10 years into a dataset with 20 return-related variables and 5 registration-related variables. After categorizing data based on North American Industry Classification System (NAICS) code, business type, filing frequency, and location features, we evaluated the statistical significance of several hypotheses. We then used feature selection together with artificial neural networks to create an audit selection predictor that achieves substantially better performance than the baseline (Fig 1). This study expands on many previous pioneering studies on machine learning and audits by increasing the explainability of the underlying models through feature selection and analysis, identifying and assessing the validity of empirical rules and hypotheses that are currently used by auditors, better partitioning the data to sub-groups that reveal latent patterns that are not accessible otherwise. A key component of our study is our use of a comprehensive, cohesive dataset with audit results that allow for validation and allows us to expand previous studies in these ways.

## 2. Background

Several data mining and machine learning methods have been proposed to address these limitations, and a multitude of case studies have been conducted regarding audit selection. Several studies contribute to the existing field of knowledge with proof-of-concept approaches to the audit selection problem. Greene et al. (1992) showed that genetic algorithms were able to effectively predict audit yields and at the time were better performing than algorithms based on statistical pattern recognition and symbolic concept acquisition [7]. Fischthal (1988) registered a patent in the United States for a neural network-based fraud detection system, which uses a clustering technique to generate a collection of classes from historical data [8]. Silva et al. (2016) conducted a case study of financial fraud using Bayesian networks on taxpayer data from Sao Paulo, Brazil [9]. The study achieved a performance rate of 41% on a dataset of 25,322 returns over 35 numeric variables. Yu et al. (2003) introduced a data mining application to detect fraud in Chinese commercial enterprises [10]. In that work, the established workflow considered domain expertise, decision tree classification, and design architecture, which resulted in an 85–90% accuracy rate for 500 businesses and 100 variables over one year. Micci-Barreca and Ramachandran (2004) conducted an audit selection case study in Texas using an SPSS-based data mining suite [11]. Compared against two other rule-based audit selection strategies in terms of sales tax adjustments, the data mining tool returned higher tax adjustments in selected audits, showing the efficacy of a machine learning approach for audit selection strategies. de Roux et al. (2018) proposed a cluster-based unsupervised learning approach for tax fraud detection on Urban Delineation tax in Colombia [12]. They evaluated their model on 1,367 tax returns with 3 variables and performed both quality review and an expert review of their model results. They found that their model accurately predicts non-underreporting returns. Other studies conducted comparative studies by which to evaluate different machine learning architectures on historical audit data. Gupta and Nagadevara (2007) performed a case study of the tradeoff between precision and sensitivity of eight developed classification models on audit selection in the value-added tax (VAT) system of India [13]. Attempting to maximize precision, they found that all models developed through data mining techniques were better than random selection. Hsu et al. (2014) conducted a pilot study of the efficiency of classification models on real-world data from the Minnesota Department of Revenue [14]. The study experimented with a combination of decision tree, Naïve Bayes, multilayer perceptron, and support vector machine classification techniques on 10,943 samples over the years 2004–2006 and achieved an increase in 63% efficiency. Results were validated by the Minnesota Department of Revenue. Rahimikia et al. (2017) investigated the effects of a hybrid model—support vector machine, multilayer perceptron, logistic regression with harmony search optimization—to detect corporate tax evasion in the food and textile financial sectors of Iran [15]. The models were tested across two different datasets with 2,000–3,000 business samples each over 21 financial variables and found that multilayer perceptron combined with harmony search optimization outperformed all other methods. Hajek and Henriques (2017) employed feature selection and classification (Bayesian belief networks, ensemble methods) for detection of financial fraud and found that interpretable Naïve Bayes outperformed all methods in terms of misclassification costs for a dataset of 622 businesses [16]. Frameworks, which incorporate domain knowledge, have been shown to increase audit selection accuracy. Wu et al. (2012) introduced a framework that utilizes association rules to minimize losses from VAT evasion in Taiwan [17]. The results show that the proposed data mining technique enhances the detection of tax evasion, and therefore can be employed to effectively reduce or minimize losses from VAT evasion. Wahab and Bakar (2021) explored a suite of machine learning algorithms for classifying income tax for the Inland Revenue Board of Malaysia [18]. The study explored the efficacy of different machine learning algorithms on audit selection classification. Ippolito and Lozano (2020) conducted a similar case study for the tax system of São Paolo [19]. They found that random forest predictors strengthened prediction results on an audit dataset over 3 years. In a similar study [20], they leveraged Sammon mapping to identify relevant dimensional data prior to training a gradient boosted tree classifier on tax audit data. Kleanthous and Chatzis (2020) applied gated mixture variational autoencoders for VAT tax audit selection [21]. They were able to show, using Cyprus tax records, that semi-supervised methods of audit selection achieve higher performance than current rule-based systems and even alternative machine learning algorithms. McKenney (2017) implemented a Return Review Program under the Internal Revenue Service to detect fraudulent returns at a lower false detection rate [22]. This pilot study was able to improve fraud detection by 59.4%.

Additionally, some work has been done surrounding compliance in the accounting and finance arena. There is a large interest in the utility and efficacy of compliance schemes, from financial reporting in Big-4 corporations [23] to investigation of IFRS policy on accounting practices [24]. Forensic accounting techniques have also become exceedingly popular in detecting noncompliant actors. Blessing (2015) evaluates the use of such techniques to curb “creative accounting” and shows the strong need of domain expertise for such techniques to remain effective [25]. Experimental methods have also long been employed to study the economic factors that influence tax compliance, as well as the impact audits have on voluntary compliance. Alm et. al. (1995) found that overweighting of low probabilities, public good provision, detection and punishment all were key determinants of compliance [26]. Niu (2011) studied the impact of New York State tax audits on voluntary compliance and found that after an audit, firms report higher sales growth rate, suggesting that audit productivity in many studies may be underestimated [27]. Gemmell and Ratto (2012) find that random audit programs provide income taxpayers with information that alters their behavioral responses to subsequent tax audits [28], thus highlighting the importance of audit selection criteria and processes. Notably, Mehdiyev et al. (2021) have explored the potential of explainability methods in the tax audit process [29]. Using prominent local and global explainability methods, the authors show that black box approaches for solving problems in public administration can be made fair and transparent.

The mentioned studies broaden the scope of machine learning methods in the tax compliance arena. Several studies illustrate the superiority of machine learning approaches over rule-based approaches, trumping auditor intuition. Comparative studies and case studies demonstrate the feasibility of machine learning in tax compliance, showing that real-world audit selection can be drastically improved if historical audit data is utilized to train effective models. Compliance studies and frameworks were constructed in attempt to create a standard for audit compliance and the audit selection process. Our study aims to build off these findings, addressing the largely ignored aspect of data compatibility. By performing rigorous statistical analysis, and assessing the validity of empirical rules used by auditors, we assured that our data was tailored for direct application of machine learning methods. We here use Artificial Neural Networks (ANNs) that can approximate any function, and hence they outperform simpler methods given enough training data, per the Universal Approximation Theorem [30]. Additionally, by conducting feature selection we identified key variables of the audit selection process.

## 3. Methods

### 3.1 Data processing

The initial dataset had 108,162 unique tax records from 10,067 businesses over 57 years. Data were organized into a matrix where each row corresponded to an anonymized business and each column corresponded to one of the 42 features (see Table SFD1 of the Data Description Sheet in S1 File). The data consisted of three data types–categorical, numeric, and datetime. Categorical data were converted to numerical data via one-hot encoding. This was done to capture information on which businesses submitted late returns. Certain features were not used in the analysis. These features are listed in S1 Table in S2 File and more information on their removal is given in Section 1.2 of S2 File. Regarding encoding, categorical data was encoded with a one-hot encoding scheme. Because the categorical features DasZipID and DasCityID were both anonymized, we were unable to extract additional information, such as latitude or longitude or census data. We note that city ID and zip code ID features were also not used during analysis, as they increase the dimensionality of the dataset and contributed to overfitting (S14 Fig). Post one-hot encoding the dataset contained 37 features. While this entire dataset was used for classification, only positive audit records were used for regression. For audit lead prediction, post-filtration (removal of features with 0 standard deviation) the dataset contained 30 features, while for audit yield prediction the dataset post-filtration contained 26 features.

Certain refinement criteria were chosen for the categorical features in our preliminary analysis. We extracted only single-location businesses that had recorded filed returns. Only businesses classified as individual, corporation, partnership, limited, and limited liability corporation were extracted, which make up 88.36% of the preprocessed dataset. The other 13.64%—joint corporations, limited liability partnerships, local government companies, organizations and trusts—were not considered. After these refinement criteria were applied, the preprocessed dataset was reduced to a total of 93,413 unique tax records from 8,646 businesses over 10 with 20 return-related variables and 5 registration-related variables. Of these businesses, only entries with nonnegative audit information were considered (510 businesses, with 6,301 tax records). We applied the analysis on this dataset, and the results are presented in S2 File, Section 2.3.2 and S10 Fig. To increase the performance and specificity of our predictive methods, we further filtered out businesses not within the 95% confidence interval for the various variables (S3 and S4 Figs). The final dataset that is used in our classification analysis presented here has 2,614 tax records from 210 unique businesses, all of them audited over the past 10 years, with 30 features total (S5 Table in S2 File). The final dataset used in our regression analysis contains 998 tax records from 76 unique businesses, audited over the past 10 years, with 26 features total (S5 Table in S2 File). Finally, quantile, min-max, z-score normalization methods were applied and evaluated during the analysis, with min-max normalization performing the best and selected for the analysis presented here (S6 Table in S2 File, S16 Fig).

### 3.2. Data visualization

**Fig 2:**
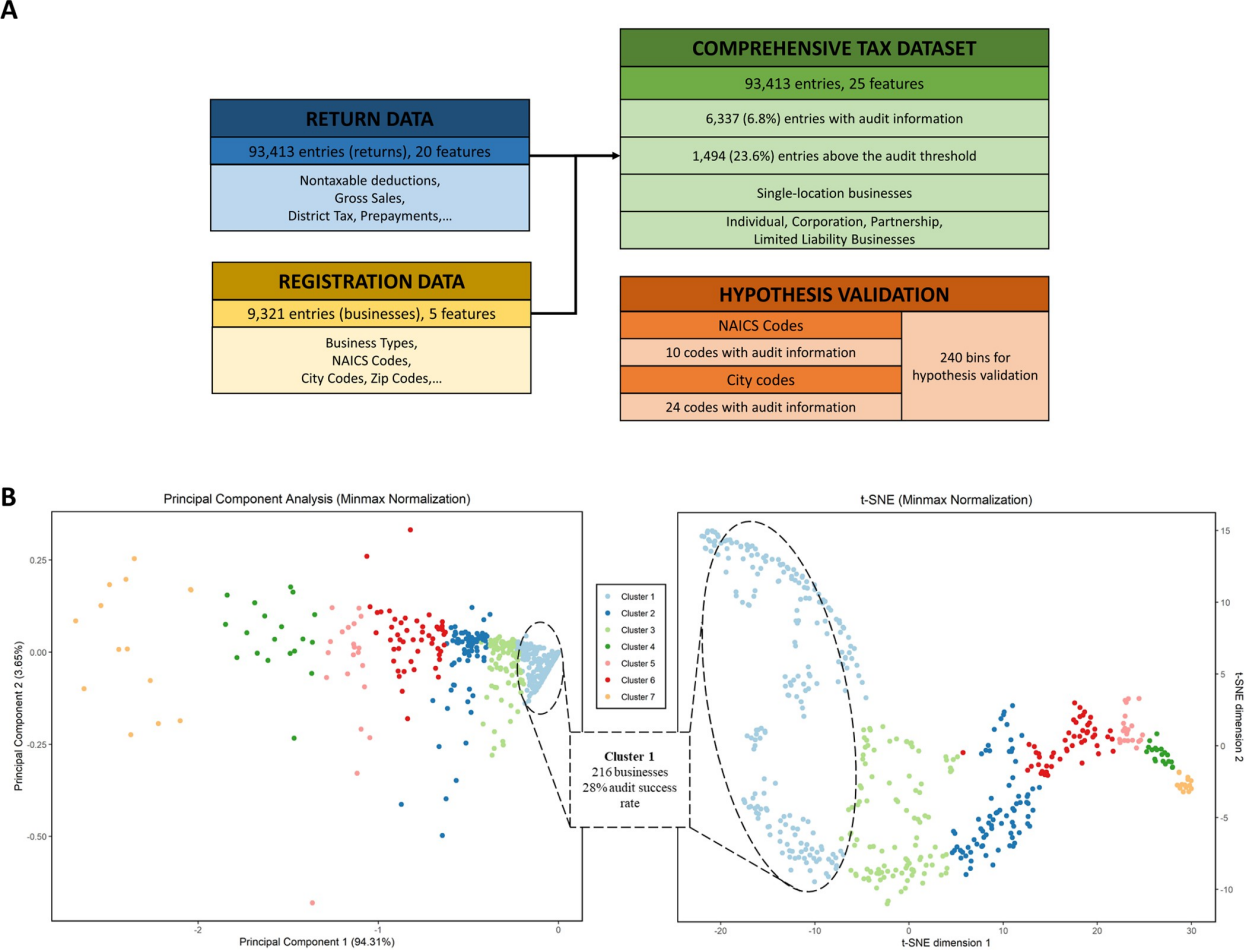
(A) Tables showing organization of the dataset, with (B) PCA and t-SNE visualization of data with minmax normalization post-filtration. K-means clustering is shown and was conducted on the PCA-data. Outliers are removed, and only datapoints with audit yield greater than $0 are shown for visualization purposes.

Principal component analysis (PCA), as well as t-Distributed Stochastic Neighbor Embedding (t-SNE) were both employed to visualize data. As several features were sparse, these features (please see Supplementary Table SFC1 of the Clustering Data Sheet in S1 File) were removed for better visualization. Additionally, data visualization with all tax record data was poor (S8 Fig), so data was first merged by business–numerical features were summed across the returns per audit period and normalized by the number of returns per audit period. Data were then hierarchically clustered for both PCA and t-SNE (Fig 2), with the elbow and gap statistic methods [31] used to identify the optimal number of clusters (S10 Fig).

### 3.3 Binning and hypothesis testing

The validity of empirical rules and other hypotheses was tested as follows. First, each business was placed on one of the 250 bins that were created based on the 10 unique North American Industry Classification System (NAICS) codes and the 25 unique City IDs in the dataset (S1 Fig). Then, various hypotheses related to gross sales, taxable sales to gross sales ratio, business classification, and late penalties were tested for different thresholds in the distribution (Tables 1 and 2). Statistical validation was performed by calculating FDR-adjusted p-values [32], and the non-parametric Wilcoxon rank-sum test.

**Table 1:** Tax audit empirical hypotheses validation.

q-value (Threshold = 0.1)	Selected Threshold for each bin (combination of CityID and NAICS)						
ORIGINAL HYPOTHESES	0.25*Mean	0.5*Mean	0.75*Mean	Mean	1.25*Mean	1.5*Mean	1.75*Mean
Reported gross sales lower than some threshold result in higher yield audits (vs. when reported gross sales is higher than the threshold)	0.11	< 10^−3^	< 10^−3^	0.10	0.15	0.16	0.20
Individual taxpayers are more likely to have positive audits compared to corporations	0.17						
The ratio of $\frac{TaxableSales}{GrossSales}$ is lower than some threshold result in higher yield audits	~ 1	~ 1	~ 1	~ 1	0.50	0.38	0.67
Variance of reported gross sales (in a specific period) lower than some threshold result in higher yield audits	0.70	~ 1	~ 1	0.79	~ 1	~ 1	~ 1
Late return penalties higher than some threshold result in higher yield audits	< 10^−6^	< 10^−6^	< 10^−5^	< 10^−5^	< 10^−6^	< 10^−4^	< 10^−3^

**Table 1**: Wilcoxon-ranksum test applied to the hypotheses. A result less than 0.1 signifies a statistically significant result. Grayed-out cells mark non-significant results. “~ 1” results denote probabilities arbitrarily close to 1.

**Table 2:** Tax audit empirical inverse hypotheses validation.

q-value (Threshold = 0.1)	Selected Threshold for each bin (combination of CityID and NAICS)						
INVERSE HYPOTHESES	0.25*Mean	0.5*Mean	0.75*Mean	Mean	1.25*Mean	1.5*Mean	1.75*Mean
Reported gross sales higher than some threshold result in higher yield audits (vs. when reported gross sales is lower than the threshold)	~ 1	~ 1	~ 1	~ 1	~ 1	~ 1	~ 1
Individual taxpayers are less likely to have positive audits compared to corporations	0.84						
The ratio of $\frac{TaxableSales}{GrossSales}$ is higher than some threshold results in higher yield audits	0.29	0.43	0.12	~ 1	~ 1	~ 1	~ 1
Variance of reported gross sales (in a specific period) higher than some threshold result in higher yield audits	~ 1	~ 1	~ 1	~ 1	~ 1	~ 1	~ 1
Late return penalties lower than some threshold result in higher yield audits	~ 1	~ 1	~ 1	~ 1	~ 1	~ 1	~ 1

**Table** 2: Wilcoxon-ranksum test applied to the inverse hypotheses. A result less than 0.1 signifies a statistically significant result. Grayed-out cells mark non-significant results. “~ 1” results denote probabilities arbitrarily close to 1.

### 3.4 Audit lead classifier

To identify positive audit leads, we trained a feed-forward Artificial Neural Network (ANN) on the 2,614 tax records of our dataset. Random search was used to select its hyperparameters, and stratified ten-fold cross-validation was employed for training. As multiple returns in the dataset are mapped to a given business, returns were grouped before being split into training and testing sets to avoid overlap of returns in both the training and testing sets/folds. In terms of labels, audits with a yield above the specified dollar threshold were classified as positive, while those with less than the threshold were classified as negative, as per CDTFA instructions on the cost of conducting an audit. The final architecture had 30 input nodes, 2 hidden layers with 19 and 8 nodes respectively, and 1 output node. The sigmoid function was used as an activation function for the nodes in each layer. The data was split 90%-10% between training/testing and validation sets. There was a high-class imbalance, with only 23% of labels being positive, hence over-sampling of minority and under-sampling of the majority class was performed [33]. Each fold was trained over 1000 epochs with a batch size 32, the Adam optimizer and binary cross-entropy as the loss function [34]. A dropout of 0.3 and L2 regularization was used to reduce overfitting. For early stopping, a delta of 0.0001 and patience, the number of epochs with no improvement after which training is stopped, of 10 was used [35].

### 3.5 Audit yield regressor

To identify the expected return of an audit, we created an audit yield regressor for positive audits. Due to the fragmentation of the dataset, using the same predictor approach as a classification for this task led to low regression performance (S17 Fig). For that reason, we grouped audit cases based on the validated hypothesis rules and categorical variables (S2 File, Section 2.4.2, S18 and S19 Figs). Extreme gradient boosted trees were trained on each case subset, and hyperparameters were selected by random search. We used ten-fold cross-validation for training, with the same 90%-10% split between training/testing and validation sets. We also had returns grouped by the business before being split into training and testing sets to avoid overlap of returns in both the training and testing sets/folds. As the audit yield is the same for multiple returns, each regressor was evaluated based on the prediction mean for a given business. The best performing model used a learning rate of 1, using 50 trees. Each tree had a max depth of 2 with 20% of the samples and 30% of features in each training set used per tree. An L1 regularization value of 4 and a linear loss function was used. Recursive Feature Elimination (RFE) was used for feature selection [36].

## 4. Results

### Dimensionality reduction reveals higher audit yields clusters and underlying rules

The dataset includes a variety of information pertaining to location, business type, audit, and return history (Fig 2A). Dimensionality reduction and subsequent visualization reveal clusters that have statistically significant over-representation of positive (or negative) audits results. Further dissecting those clusters uncovers the rules that are common to their members. For example, cluster 1 was the cluster with the highest audit success rate (27.8% vs. 19.5% which is the average). A list of those clusters and underlying rules can be found in Supplementary Table SFC3 of the Clustering Data Sheet in S1 File, while S11 and S12 Figs depict such clusters with different normalization schemas. Considering all returns and features, and by ordering the numerical features for each return, patterns became evident. For example, pattern 24 in Supplementary Table SFC4 of the Clustering Data Sheet in S1 File contains 1,290 returns, with 141 of them been audited. In these 141 returns, 116 (82.2%) of them contained a positive audit yield and 97 (68.8%) of them contained audit yield greater than the threshold, a significantly higher success rate for selecting a non-zero audit yield (82.2% vs. 33% on average, p-value = 3.8∙10^−35^) or a positive audit, i.e. one with a yield higher than the threshold (68.8% vs. 23.6% on average, p-value = 1.78∙10^−31^). In addition, the audit ratio (141/1290 = 11%) is also significantly higher than the overall audit ratio (6,301/93,413 = 6.75%), which means that the audits also showed returns with this specific pattern. This analysis enabled us to not only visualize the dataset but also investigate particular patterns that emerged outside of our provided feature set. Observing patterns in high-yield audited returns can enable the CDTFA the ability to develop key identifiers for businesses that file their returns in this way. Particularly, it may be possible after this analysis to create synthetic features that represent the significance captured by these patterns.

### Empirical based hypotheses validated by data

**Fig 3:**
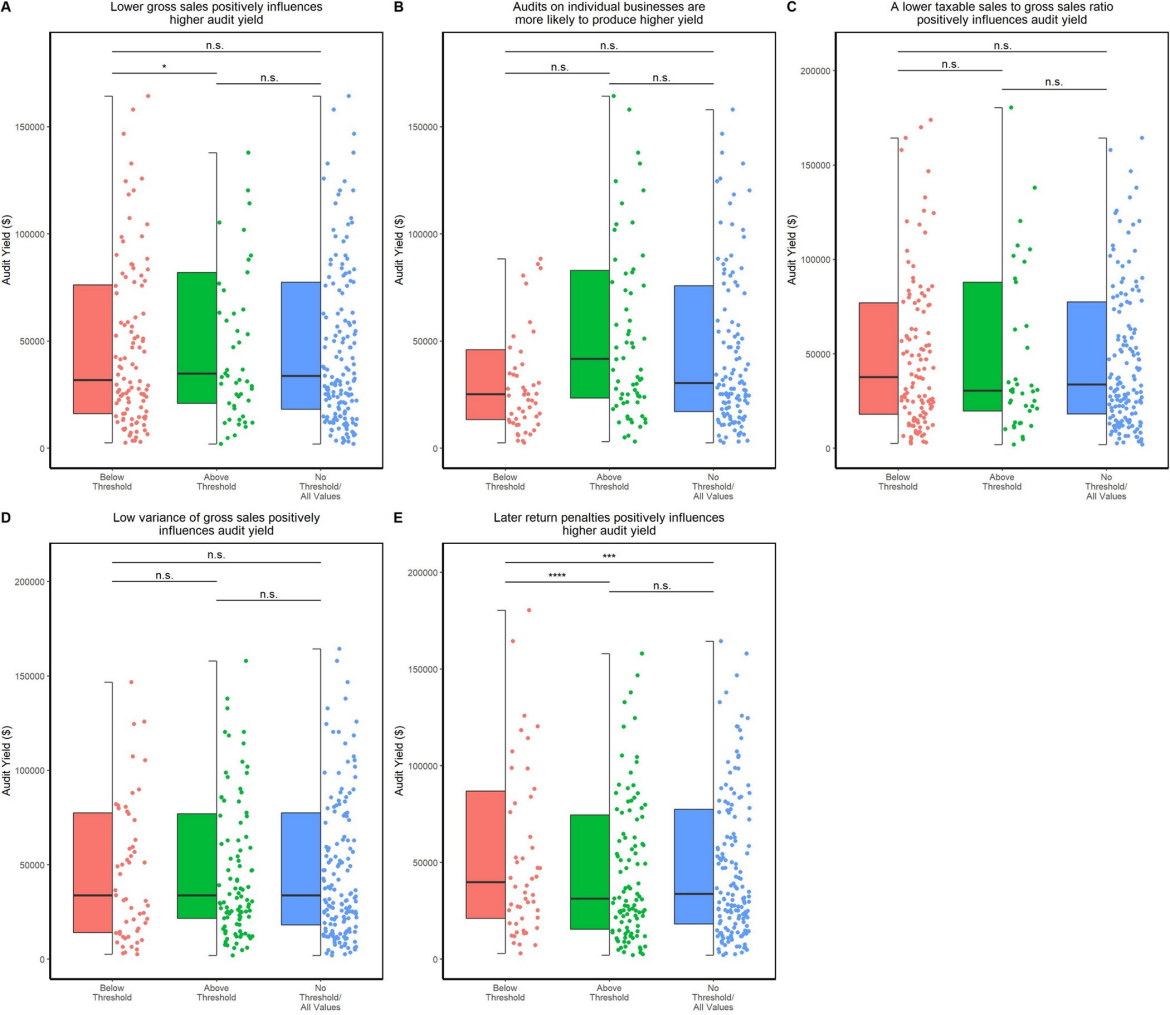
Statistical validation using Wilcoxon-ranksum test of all five hypotheses. Distribution of data for each threshold bin are plotted with statistical significance denoted above each pair of groups. * denotes a p-value less than 0.1, ** a p-value less than 0.05 and *** a p-value less than 0.001. n.s. denotes a non-significant result. For all graphs above, the threshold was set to the mean.

Next, we investigated the rules by which tax departments employ an in-house method to detect anomalies on tax reports and select businesses to audit. Table 1 depicts five such rules that are empirically chosen and are used in practice. We evaluated these hypotheses on varying thresholds depending on the mean of the respective bin (see Methods). Fig 3 presents a visual representation with boxplots in the cases where the threshold is equal to the mean of the distribution for the respective bin. Our results show that two out of the five hypotheses pan out for a given value of the threshold. The higher propensity of positive audits was recorded when the reported gross sales are lower than the mean and when there have been late return penalties. Interestingly, the ratio of taxable vs. gross sales is not a good indicator of audit success, and only those businesses with no variance in their reported gross sales had a statistically significant over-representation of positive audits. This was an unexpected result, as the taxable to gross sales ratio is usually employed by auditors to identify businesses with an excessive percentage of non-taxable sales within the same or similar type of business activity i.e. in certain industries where it is not common to claim deductions, this indicator serves to flag a disproportionate amount of uncommon deductions. It is possible that due to the underrepresentation of businesses in these industries in our dataset that this hypothesis was not found significant. We also tested the inverse hypotheses, which were all rejected as expected (Table 2). Examining these hypotheses gave us insight as to whether auditor intuition was reflected in the dataset we were provided with, as well as ideas on what features to look for from an explainability standpoint when constructing our predictors.

### 65% of the audit leads produced by the classifier are positive

**Fig 4:**
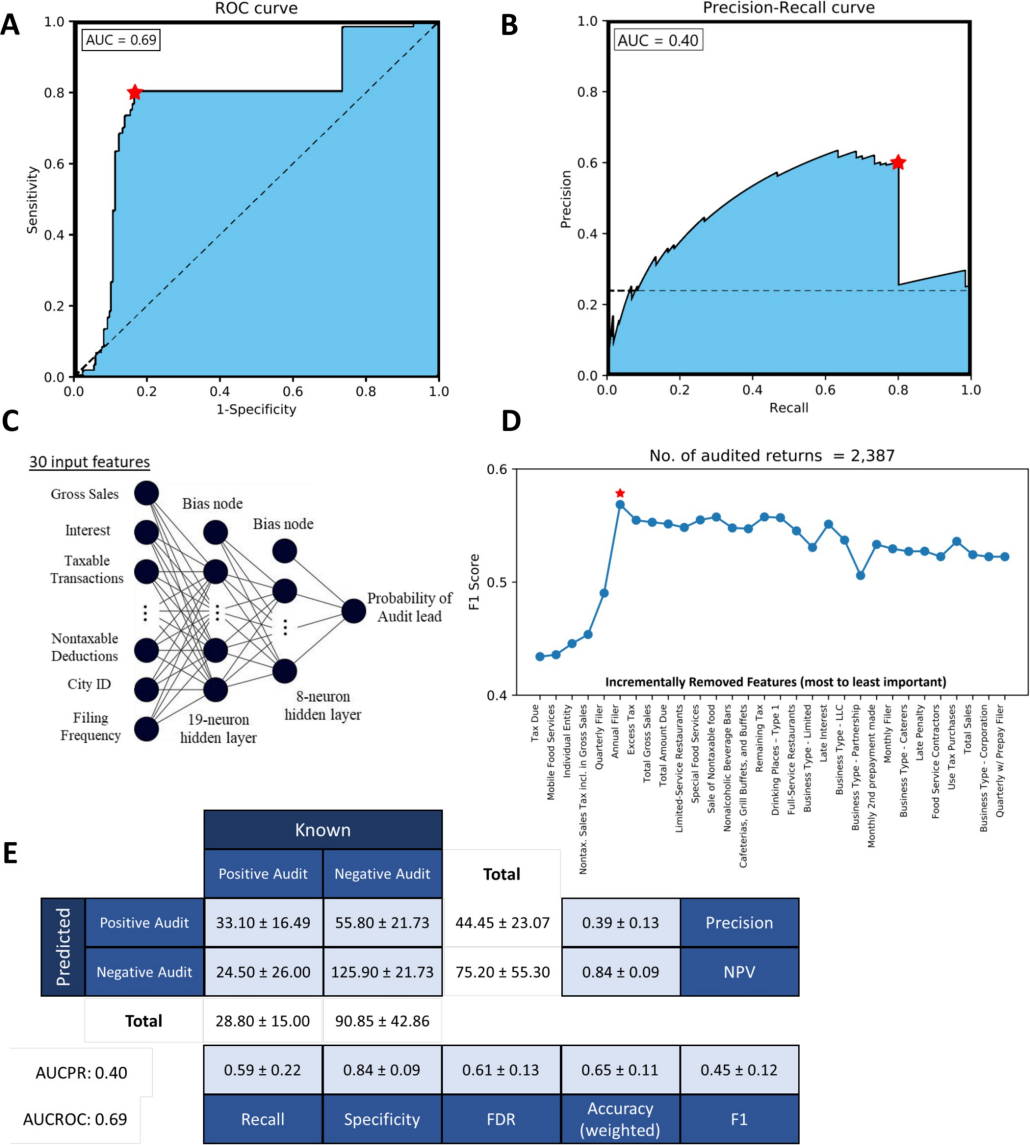
(A) Receiver operating characteristic (ROC) curve, (B) precision-recall curve, and (C) audit lead classifier architecture with (D) recursive feature elimination results. The red star signifies the feature added after which model performance no longer improves. (E) displays the confusion matrix for the trained classification model. The average F_1_ score for the positive class was 0.45 across all 10 folds, with AUROC and AUPRC being 0.86 and 0.47, respectively.

The audit lead classifier was able to achieve average training and testing accuracies of 65.3% and 63.6% across all ten folds, while on the validation set it achieved an accuracy of 61.2%, and F1 score of 0.42, with precision and recall of 40.1% and 58.7%, respectively. Fig 4 shows the architecture, ROC curve, precision-recall curve, and confusion matrix for the audit lead classifier. Additionally, running RFE on the audit lead predictor revealed some unexpected features being selected, such as the tax amount due, the type of the business (mobile food or individually owned), the amount of sales tax included in gross sales, and whether the business owner filed their taxes quarterly or annually. Fig 4D shows how the removal of each feature impacts the performance of the classifier, as a function of the F1 score.

### 30% of all variation in audit yield can be measured by an audit yield regressor

**Fig 5:**
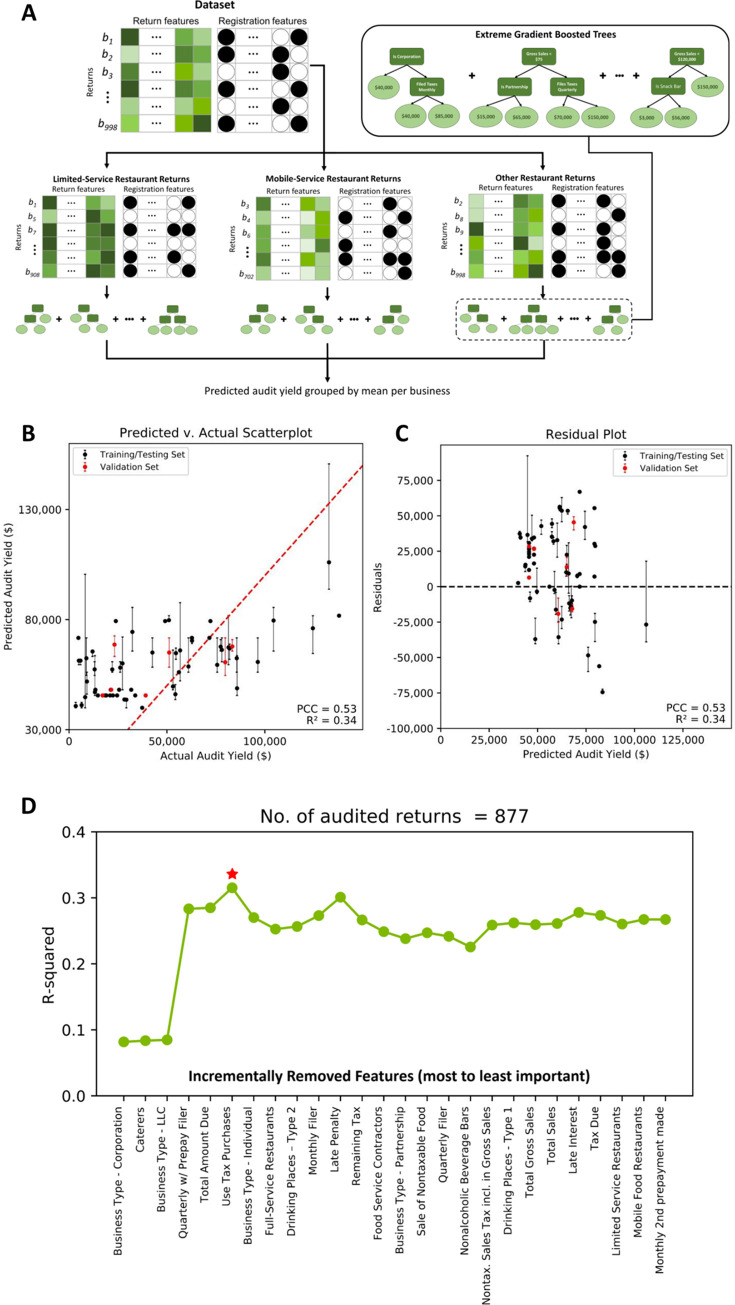
(A) Regressor architecture and pipeline, (B) predicted vs. actual and (C) residual plots. Error bars represent the prediction on multiple returns, where each point represents the mean of predictions for a single business. Two outlying points with audit yield greater than $20,000 were removed for better visualization. (D) shows recursive feature elimination results for the trained regression model. The red star signifies the feature added after which model performance no longer improves.

Next, we investigated whether we could predict the actual amount of a positive audit, given its characteristics. After experimenting with several binning scenarios and regression techniques, we concluded that binning based on the validated hypotheses together with a boosting tree method produces optimal results (see Methods). The selected audit yield regressor achieved an average training and testing R-squared of 0.34 and 0.32 across all ten folds and achieved an R-squared of 0.34 on the validation set. Fig 5 shows the architecture and pipeline, predicted v. actual scatterplot, and a residual scatterplot for the selected NAICS code-based audit lead regressor. Similar to the classifier, running RFE on the audit lead predictor revealed that the important features are the type of the business, catering option, pre-paying and filing characteristics, the amount due, and its use tax purchases (Fig 5D).

It is important to note that while artificial neural networks allow us to extract performance and accuracy from our data, their inner-workings are opaque. By performing feature selection via methods like RFE, we were able to grasp a greater understanding of the impact of variables in our feature set on the prediction task at hand. This knowledge can be instrumental the CDTFA, as it can help them optimize their audit selection criteria.

## Discussion

This work is building upon decades of empirical tax audit selection and attempts to use computational methods to automate pattern analysis and outlier detection. There are several lessons that we learned on this journey. First, there is a need for standardization, integration, and preprocessing of tax records so that advanced machine learning techniques can be applied. Although this need is less dire than other domains, such as life sciences or business development, due to the digitization and nature of the tax records, we have encountered a number of issues, ranging from unmappable audit yield to audit period timestamps and lack of a large enough pool of accounts that were audited to data anonymization and lack of access to dark pools of data that contain important features for building a positive audit predictor.

Another observation is that the current process is distributed, subjective, and less driven by data analysis. Although there are tools that analyze data for identifying outliers and potential leads, these tools are usually limited in both scope and capabilities, mostly relying on decision trees and simple regression models. Usually, empirical rules are subjective and have never been subjected to statistical tests to assess their validity. Our analysis shows that, as expected, a small subset of features is sufficient to achieve accurate audit lead prediction, although what these features are and the how the methodology needs to be refined to broaden its scope and accuracy, is work in progress. Given the revolution in machine learning that we have witnessed the past decade and the plethora of data that tax departments have in their disposal, it creates a perfect storm for a disrupting technology such as deep learning to operate on the variety of contextual data that are currently available to achieve precision taxation and compliance at scale.

Future work includes the amelioration of both data and computational resources. In terms of data, we will expand the scope of this work to other sectors beyond restaurants, and other districts beyond Northern California. That would lead to generalizable methods that may consist of a core module that can be sector and location agnostic, and specialized learners that fine-tune predictions based on those characteristics. It seems worth noting that the data restriction to nonnegative audit information could be expanded should more business and audit information be obtained. It seems prudent that in a continued collaboration with the CDTFA, we be able to conduct actual audits based on criteria selected by our methods to validate the findings of this study. In terms of computational methods, we will work on adopting time series techniques, such as recurrent neural networks and Long Short-Term Memory (LSTM) architectures, as well as the integration of multiple other sources of information, coming from employment, property, and income tax records, as well as receipts or similar non-tax information. As filtration removed many tax records above the 95% confidence interval, it would be interesting performing a similar analysis on these data points. Considering features from multiple data streams would allow our models to make more informed predictions. Additionally, techniques that increase the explainability and trustworthiness of the results would be helpful, as well as a more in-depth look at the candidate business patterns to understand why they have been selected should be done.

Finally, a key topic that was not addressed here, but it is of high importance is algorithmic bias. From dataset population bias to aggregation bias, it is important to take steps to assess and improve fairness to ensure our predictive models achieve non-discriminatory results. Steps such as better preprocessing and adding fairness constraints, as well as introducing fairness penalties to our predictive models would be of great interest. The work here and extensions we propose above will pave the way and contribute towards a better, more precision and fair tax system that will adapt to new developments and events while adopting an evidence-based approach to public service.

## Supporting information

S1 Fig Possible values for the categorical variables in Registration Data–the number of unique values is shown for City ID and Zip ID. (TIF)

S2 Fig Heatmap showing clusters based on Pearson correlation between the various return data features. Clusters with Pearson correlation greater than 0.9 are colored both in label and dendrograms.
(TIF)

S3 Fig Data distribution of Total Gross Sales prior to filtration. Non-normalized (top left), min-max normalization (top right). quantile normalization (bottom left), and z-score normalization (bottom right) are shown above, with red and blue dots representing 95% confidence interval and mean respectively. Inset plots show the entire dataset while the main plots show the inset plots zoomed in to the 95% confidence interval range.
(TIF)

S4 Fig Data distribution of Total Gross Sales post-filtration. Non-normalized (top left), min-max normalization (top right). quantile normalization (bottom left), and z-score normalization (bottom right) are shown above, with red and blue dots representing 95% confidence interval and mean respectively. Inset plots show the entire dataset while the main plots show the inset plots zoomed in to the 95% confidence interval range.
(TIF)

S5 Fig PCA visualization of all 93,413 returns without filtration. Non-normalized (top left), min-max normalization (top right). quantile normalization (bottom left), and z-score normalization (bottom right) are shown above. The returns with different audit outcome are in different color (black: Not being audited; blue: Being audited but get zero audit yield; red: Being audited with audit yield greater than zero; green: Being audited with audited yield greater than CDTFA’s threshold).
(TIF)

S6 Fig t-SNE visualization of all 93,413 returns without filtration. Non-normalized (top left), min-max normalization (top right). quantile normalization (bottom left), and z-score normalization (bottom right) are shown above. The returns with different audit outcome are in different color (black: Not being audited; blue: Being audited but get zero audit yield; red: Being audited with audit yield greater than zero; green: Being audited with audited yield greater than CDTFA’s threshold).
(TIF)

S7 Fig Visualization of all 93,413 returns without filtration (with feature-wise quantile normalization) by (A) PCA and (B) t-SNE. The returns with different audit outcome are in different color (black: Not being audited; blue: Being audited but get zero audit yield; red: Being audited with audit yield greater than zero; green: Being audited with audited yield greater than CDTFA’s threshold). (C) shows the t-SNE plot of 93,413 feature-wise quantile normalized returns. The corresponding to the eight largest clusters of feature order are in represented by different colors.
(TIF)

S8 Fig Elbow method (top left) and gap statistic method (top right) conducted on result of hierarchical clustering performed on min-max normalized PCA data. Two clusters were selected as optimal. PCA (bottom left) and t-SNE (bottom right) visualization of the clustered data with min-max normalization post-filtration.
(TIF)

S9 Fig Elbow method (top left) and gap statistic method (top right) conducted on result of hierarchical clustering performed on audit period-merged min-max-normalized PCA data. Two clusters were selected as optimal. PCA (bottom left) and t-SNE (bottom right) visualization of the clustered data with min-max normalization post-filtration.
(TIF)

S10 Fig Elbow method (top left) and gap statistic method (top right) conducted on result of hierarchical clustering performed on audit period-merged min-max-normalized PCA data (without outliers). Seven clusters were selected as the optimal.
(TIF)

S11 Fig Elbow method (top left) and gap statistic method (top right) conducted on result of hierarchical clustering performed on audit period-merged min-max-normalized t-SNE data. Six clusters were selected as the optimal. PCA (bottom left) and t-SNE (bottom right) visualization of the clustered data with min-max normalization post-filtration.
(TIF)

S12 Fig Elbow method (top left) and gap statistic method (top right) conducted on result of hierarchical clustering performed on audit period-merged z-score-normalized PCA data. Two clusters were selected as optimal. PCA (bottom left) and t-SNE (bottom right) visualization of the clustered data with z-score normalization post-filtration.
(TIF)

S13 Fig Elbow method (top left) and gap statistic method (top right) conducted on result of hierarchical clustering performed on audit period-merged quantile-normalized PCA data to find the number of clusters. Six clusters were selected as the optimal. PCA (bottom left) and t-SNE (bottom right) visualization of the clustered data with quantile normalization post-filtration.
(TIF)

S14 Fig Accuracy (top left) and loss per epoch (top right) graphs for a single cross-validation fold using data with all 135 features exhibiting overfitting, as well as ROC (bottom left) and precision-recall (bottom right) for the validation set.
(TIF)

S15 Fig (A) Receiver operating characteristic and (B) precision-recall curves for audit lead classifier without filtration of returns outside of the 95% confidence interval with (C) confusion matrix for classifier trained without filtration of returns outside of the 95% confidence interval.
(TIF)

S16 Fig Receiver operating characteristic (ROC) and precision-recall (PR) curves for classifier trained with z-score normalized data (top) and ROC and PR curves for classifier trained with quantile-normalized data (bottom).
(TIF)

S17 Fig Predicted v. Actual (left) and Residual (right) plots for single regressor model. Error bars represent the prediction on multiple returns, where each point represents the mean of predictions for a single business.
(TIF)

S18 Fig Predicted v. Actual (left) and Residual (right) plots ensemble model created with (A) Hypothesis 1 splits, and (B) Hypothesis 5 splits. Error bars represent the prediction on multiple returns, where each point represents the mean of predictions for a single business.
(TIF)

S19 Fig Predicted v. Actual (left) and Residual (right) plots ensemble model created with splits on business type. Error bars represent the prediction on multiple returns, where each point represents the mean of predictions for a single business.
(TIF)

S1 File Supporting File containing data feature description and clustering information. (XLSX)

S2 File Supplementary materials and supporting tables. (DOCX)
