# Supplementary figures and images for: Audit lead selection and yield prediction from historical tax data using artificial neural networks
Source: PLoS One. 2022 Nov 30;17(11):e0278121. doi: 10.1371/journal.pone.0278121 (PMC9710839; doi:10.1371/journal.pone.0278121)

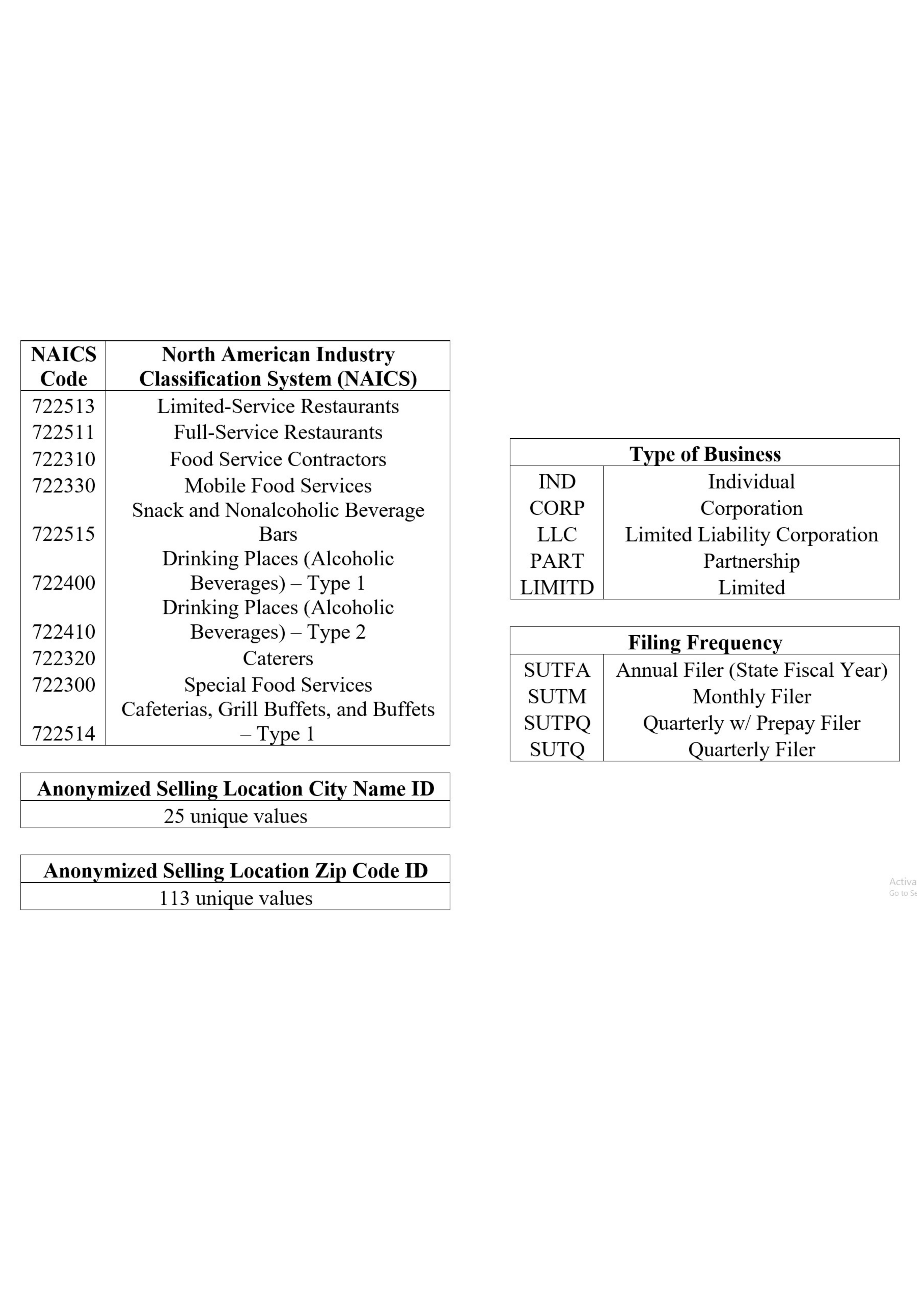

Supplement: S1 Fig — (TIF) [file pone.0278121.s001.tif]

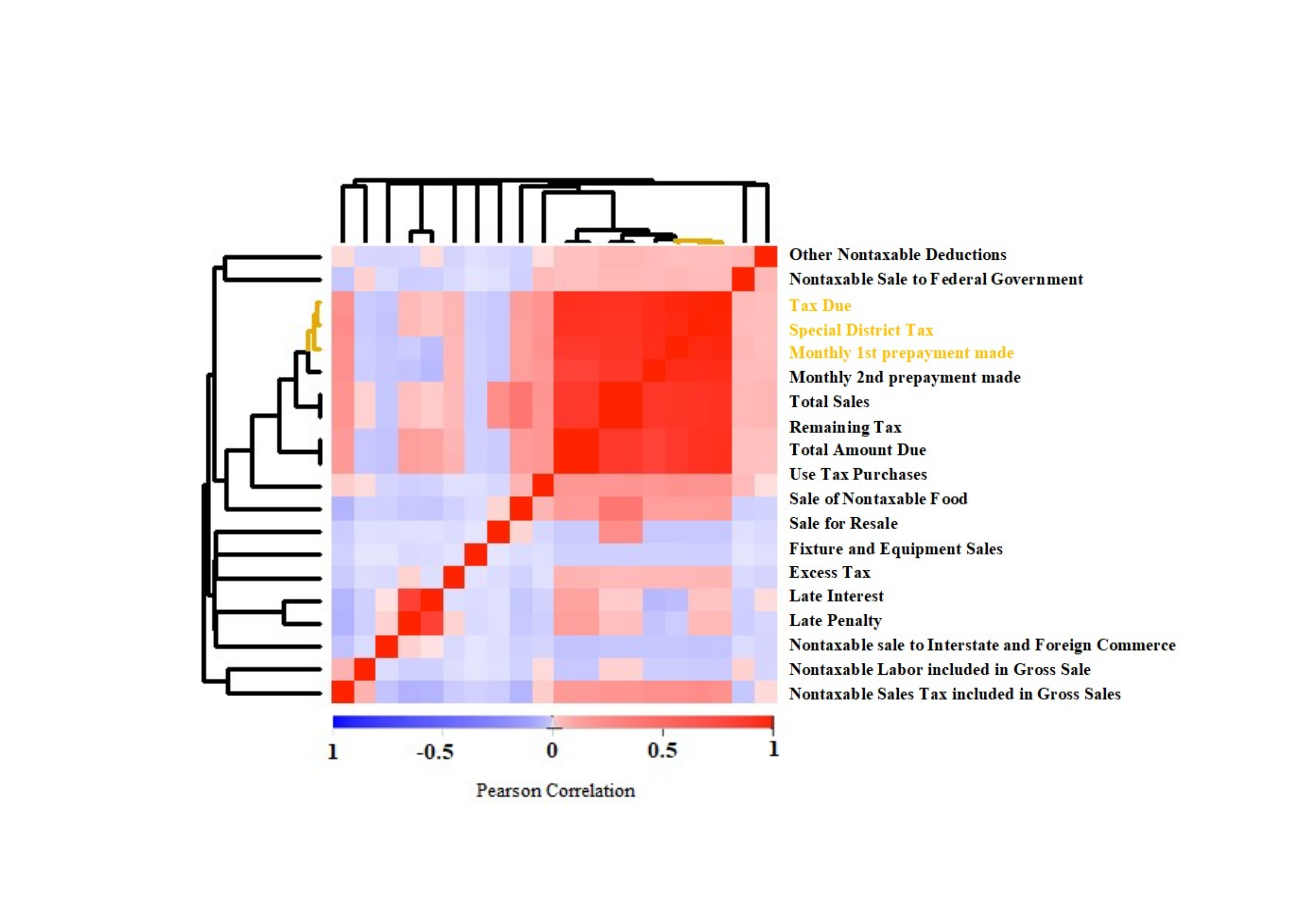

Supplement: S2 Fig — Clusters with Pearson correlation greater than 0.9 are colored both in label and dendrograms. (TIF) [file pone.0278121.s002.tif]

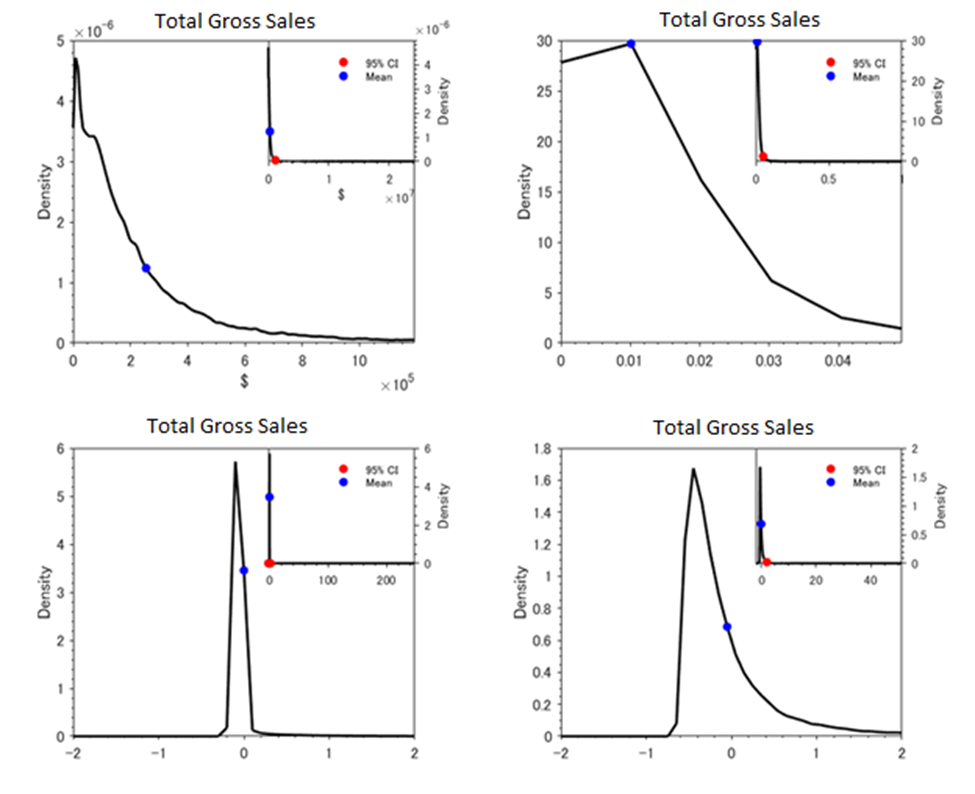

Supplement: S3 Fig — Non-normalized (top left), min-max normalization (top right). quantile normalization (bottom left), and z-score normalization (bottom right) are shown above, with red and blue dots representing 95% confidence interval and mean respectively. Inset plots show the entire dataset while the main plots show the inset plots zoomed in to the 95% confidence interval range. (TIF) [file pone.0278121.s003.tif]

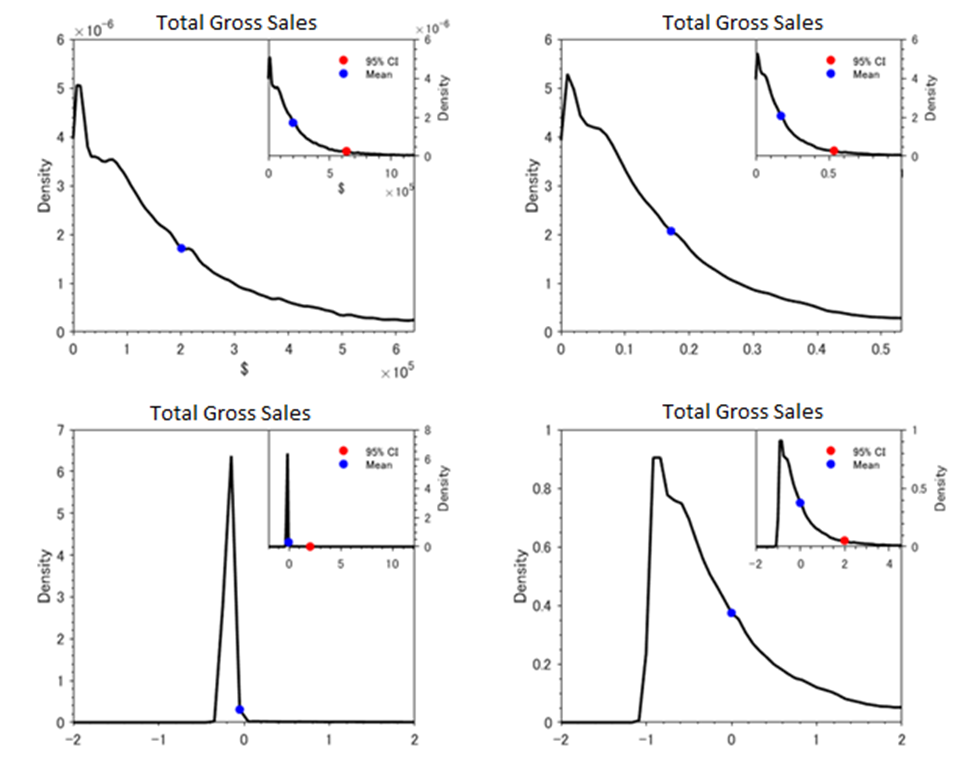

Supplement: S4 Fig — Non-normalized (top left), min-max normalization (top right). quantile normalization (bottom left), and z-score normalization (bottom right) are shown above, with red and blue dots representing 95% confidence interval and mean respectively. Inset plots show the entire dataset while the main plots show the inset plots zoomed in to the 95% confidence interval range. (TIF) [file pone.0278121.s004.tif]

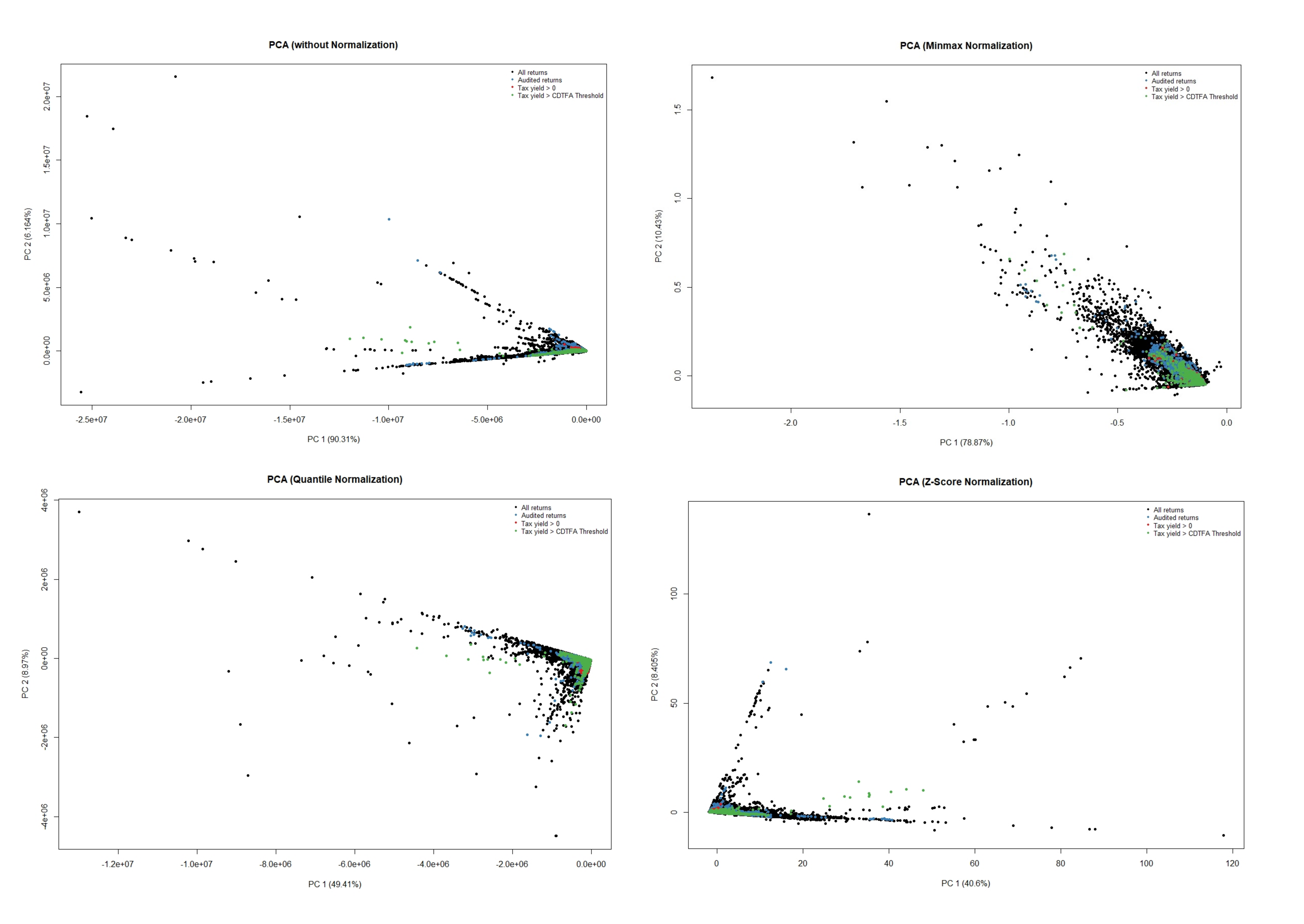

Supplement: S5 Fig — Non-normalized (top left), min-max normalization (top right). quantile normalization (bottom left), and z-score normalization (bottom right) are shown above. The returns with different audit outcome are in different color (black: Not being audited; blue: Being audited but get zero audit yield; red: Being audited with audit yield greater than zero; green: Being audited with audited yield greater than CDTFA’s threshold). (TIF) [file pone.0278121.s005.tif]

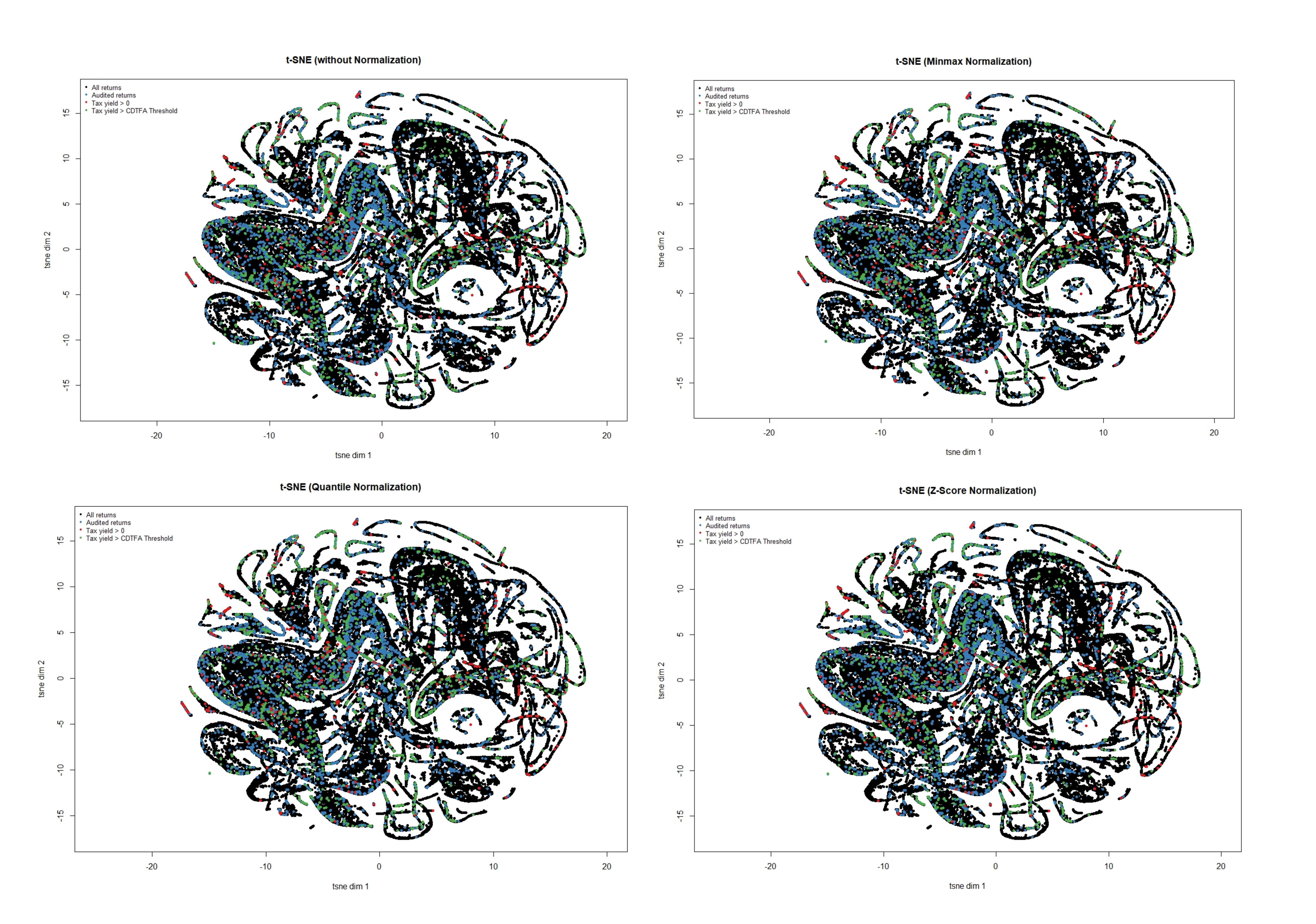

Supplement: S6 Fig — Non-normalized (top left), min-max normalization (top right). quantile normalization (bottom left), and z-score normalization (bottom right) are shown above. The returns with different audit outcome are in different color (black: Not being audited; blue: Being audited but get zero audit yield; red: Being audited with audit yield greater than zero; green: Being audited with audited yield greater than CDTFA’s threshold). (TIF) [file pone.0278121.s006.tif]

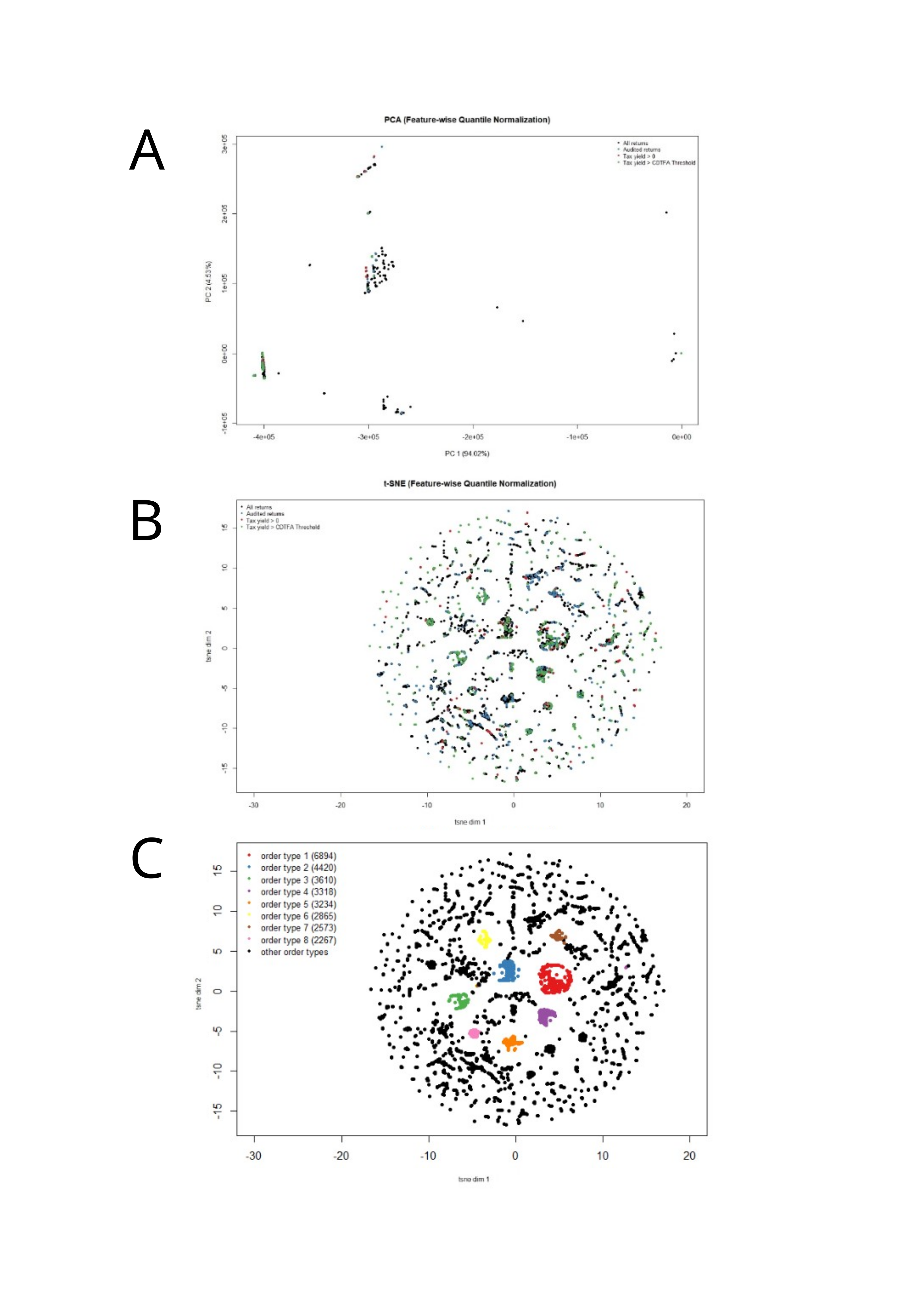

Supplement: S7 Fig — Visualization of all 93,413 returns without filtration (with feature-wise quantile normalization) by (A) PCA and (B) t-SNE. The returns with different audit outcome are in different color (black: Not being audited; blue: Being audited but get zero audit yield; red: Being audited with audit yield greater than zero; green: Being audited with audited yield greater than CDTFA’s threshold). (C) shows the t-SNE plot of 93,413 feature-wise quantile normalized returns. The corresponding to the eight largest clusters of feature order are in represented by different colors. (TIF) [file pone.0278121.s007.tif]

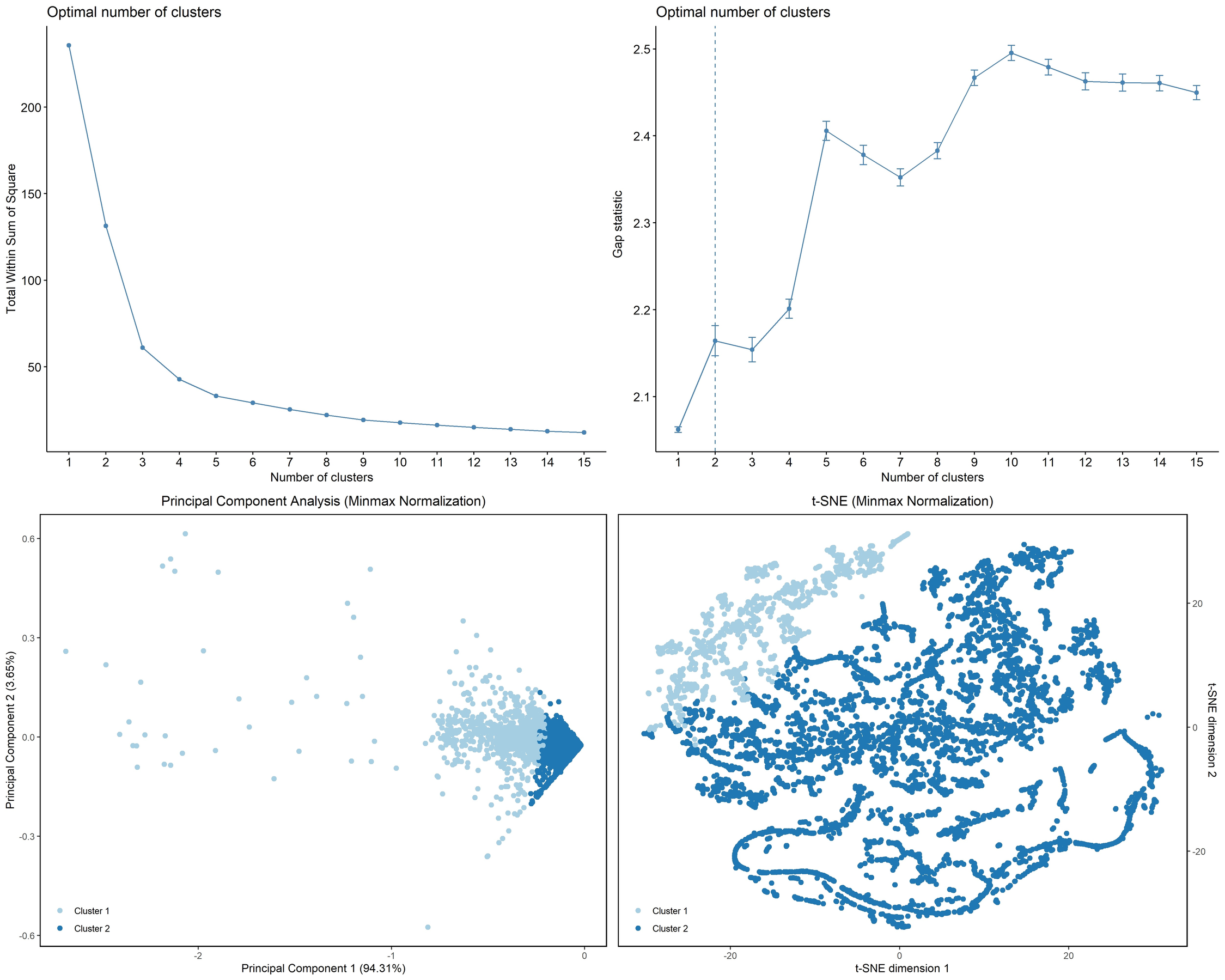

Supplement: S8 Fig — Elbow method (top left) and gap statistic method (top right) conducted on result of hierarchical clustering performed on min-max normalized PCA data. Two clusters were selected as optimal. PCA (bottom left) and t-SNE (bottom right) visualization of the clustered data with min-max normalization post-filtration. (TIF) [file pone.0278121.s008.tif]

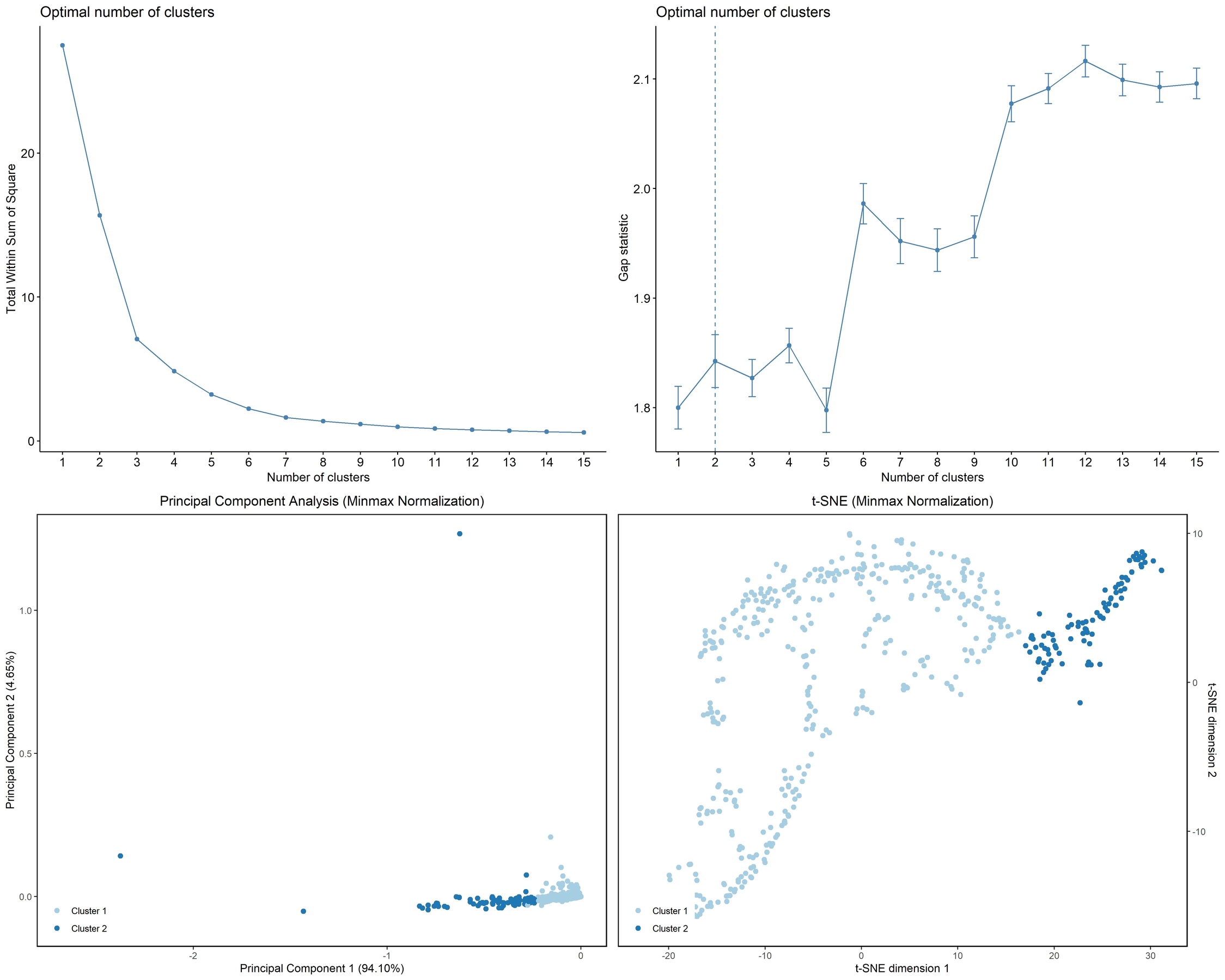

Supplement: S9 Fig — Elbow method (top left) and gap statistic method (top right) conducted on result of hierarchical clustering performed on audit period-merged min-max-normalized PCA data. Two clusters were selected as optimal. PCA (bottom left) and t-SNE (bottom right) visualization of the clustered data with min-max normalization post-filtration. (TIF) [file pone.0278121.s009.tif]

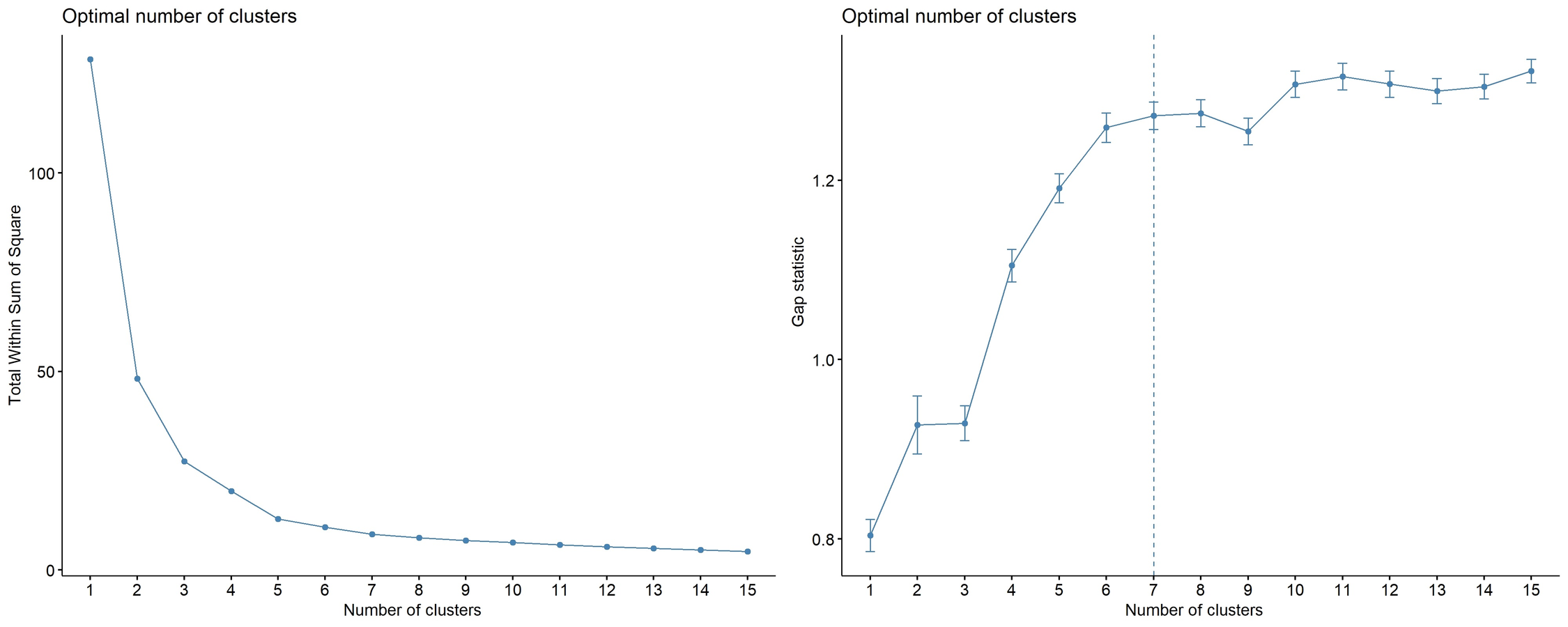

Supplement: S10 Fig — Elbow method (top left) and gap statistic method (top right) conducted on result of hierarchical clustering performed on audit period-merged min-max-normalized PCA data (without outliers). Seven clusters were selected as the optimal. (TIF) [file pone.0278121.s010.tif]

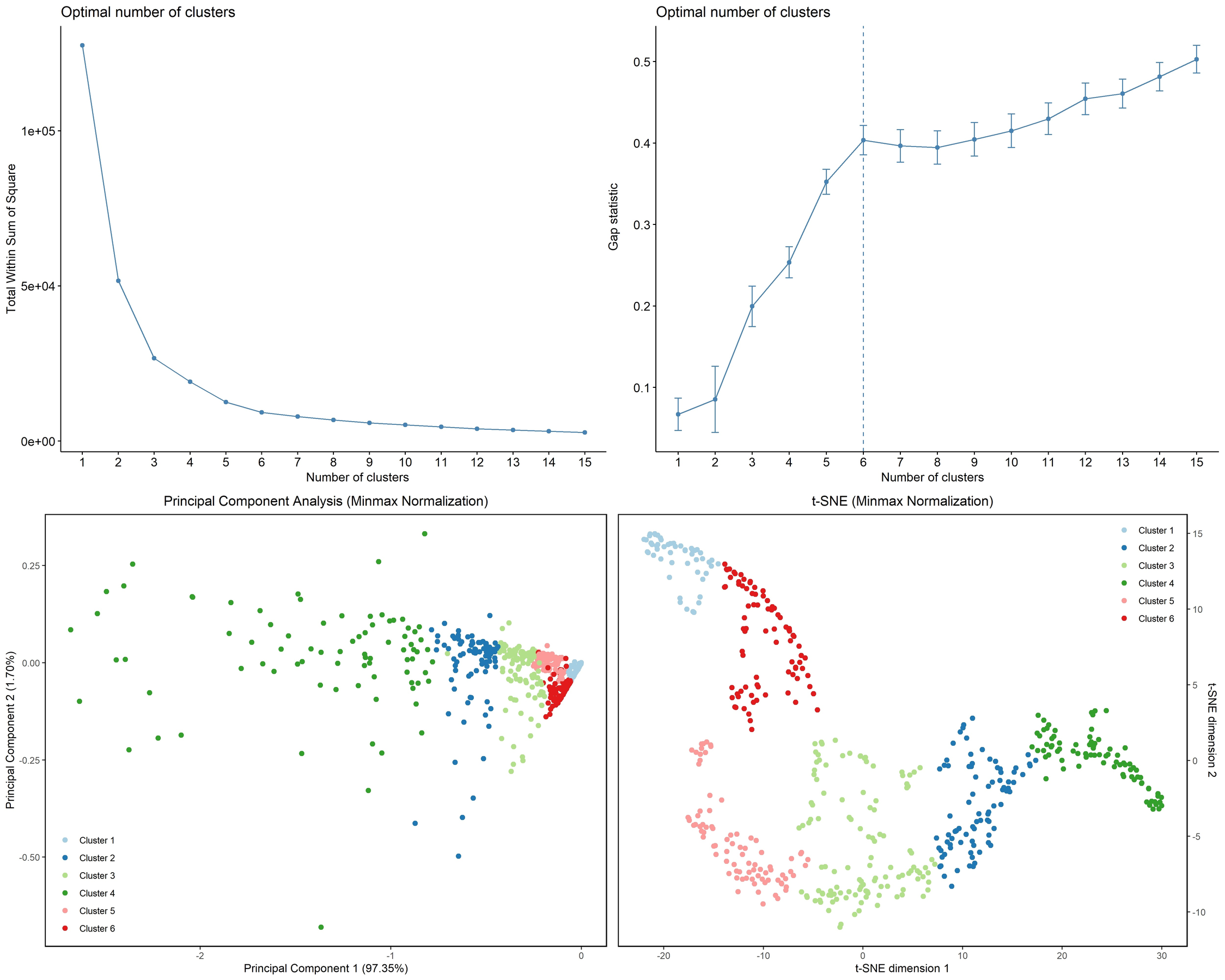

Supplement: S11 Fig — Elbow method (top left) and gap statistic method (top right) conducted on result of hierarchical clustering performed on audit period-merged min-max-normalized t-SNE data. Six clusters were selected as the optimal. PCA (bottom left) and t-SNE (bottom right) visualization of the clustered data with min-max normalization post-filtration. (TIF) [file pone.0278121.s011.tif]

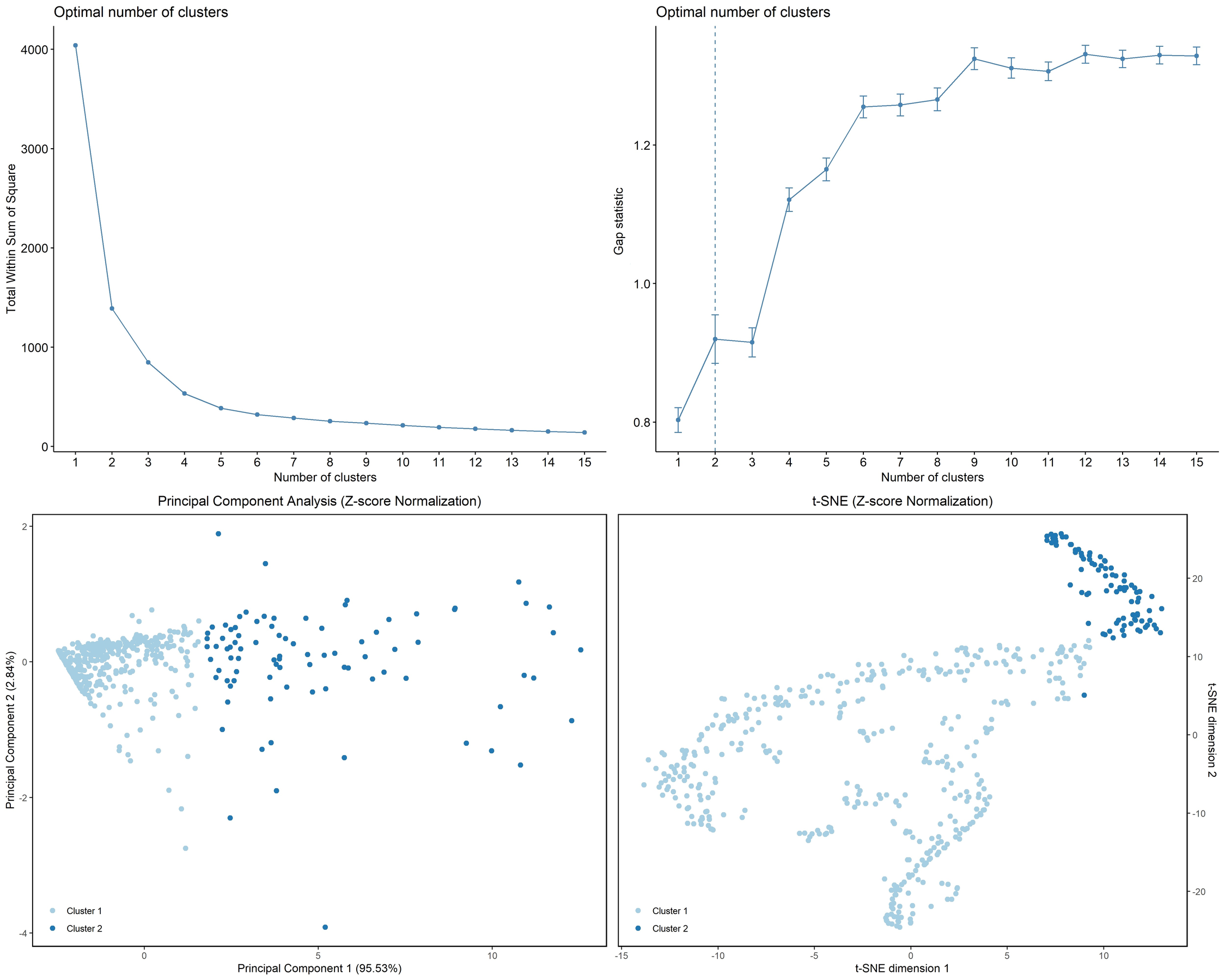

Supplement: S12 Fig — Elbow method (top left) and gap statistic method (top right) conducted on result of hierarchical clustering performed on audit period-merged z-score-normalized PCA data. Two clusters were selected as optimal. PCA (bottom left) and t-SNE (bottom right) visualization of the clustered data with z-score normalization post-filtration. (TIF) [file pone.0278121.s012.tif]

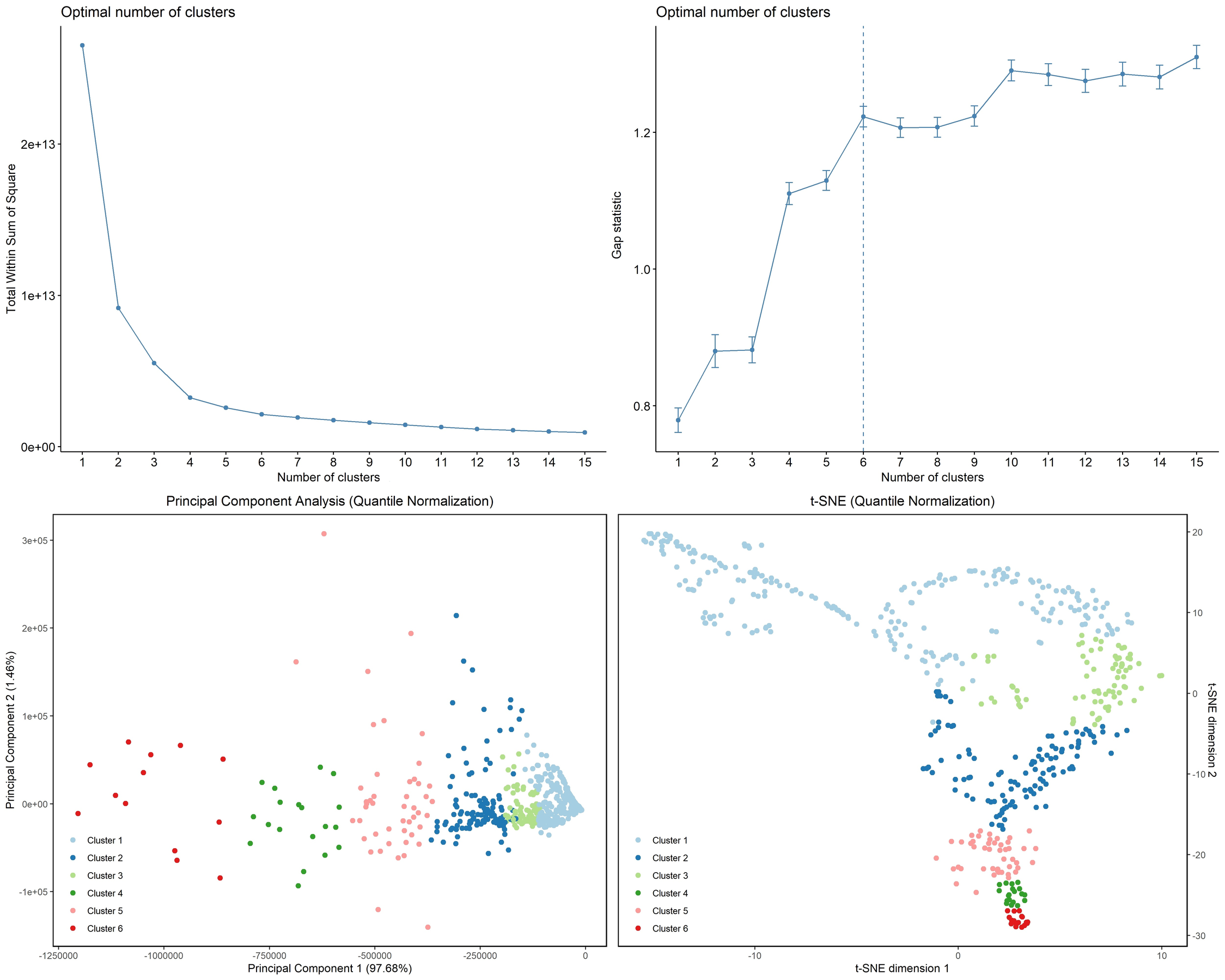

Supplement: S13 Fig — Elbow method (top left) and gap statistic method (top right) conducted on result of hierarchical clustering performed on audit period-merged quantile-normalized PCA data to find the number of clusters. Six clusters were selected as the optimal. PCA (bottom left) and t-SNE (bottom right) visualization of the clustered data with quantile normalization post-filtration. (TIF) [file pone.0278121.s013.tif]

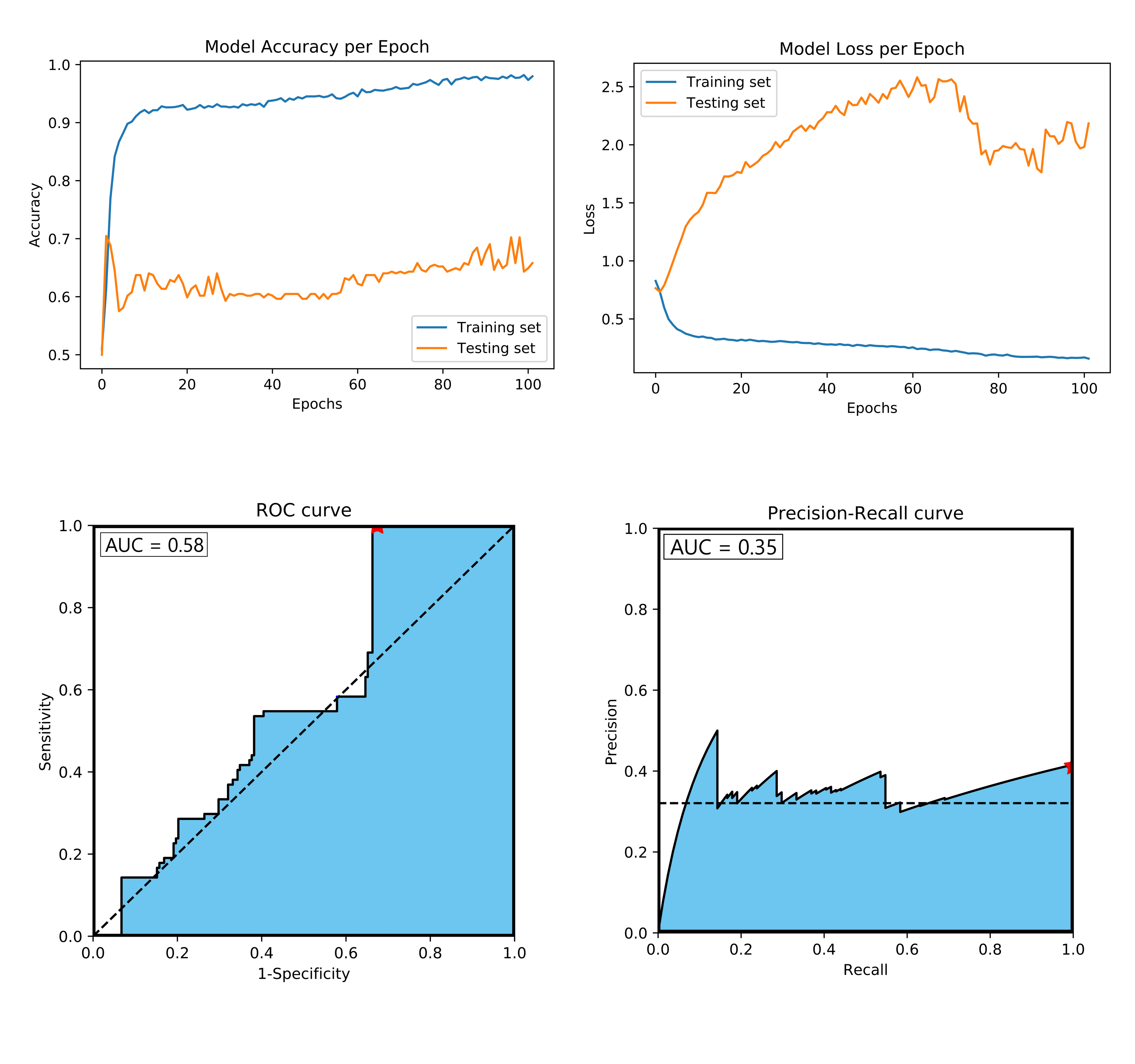

Supplement: S14 Fig — Accuracy (top left) and loss per epoch (top right) graphs for a single cross-validation fold using data with all 135 features exhibiting overfitting, as well as ROC (bottom left) and precision-recall (bottom right) for the validation set. (TIF) [file pone.0278121.s014.tif]

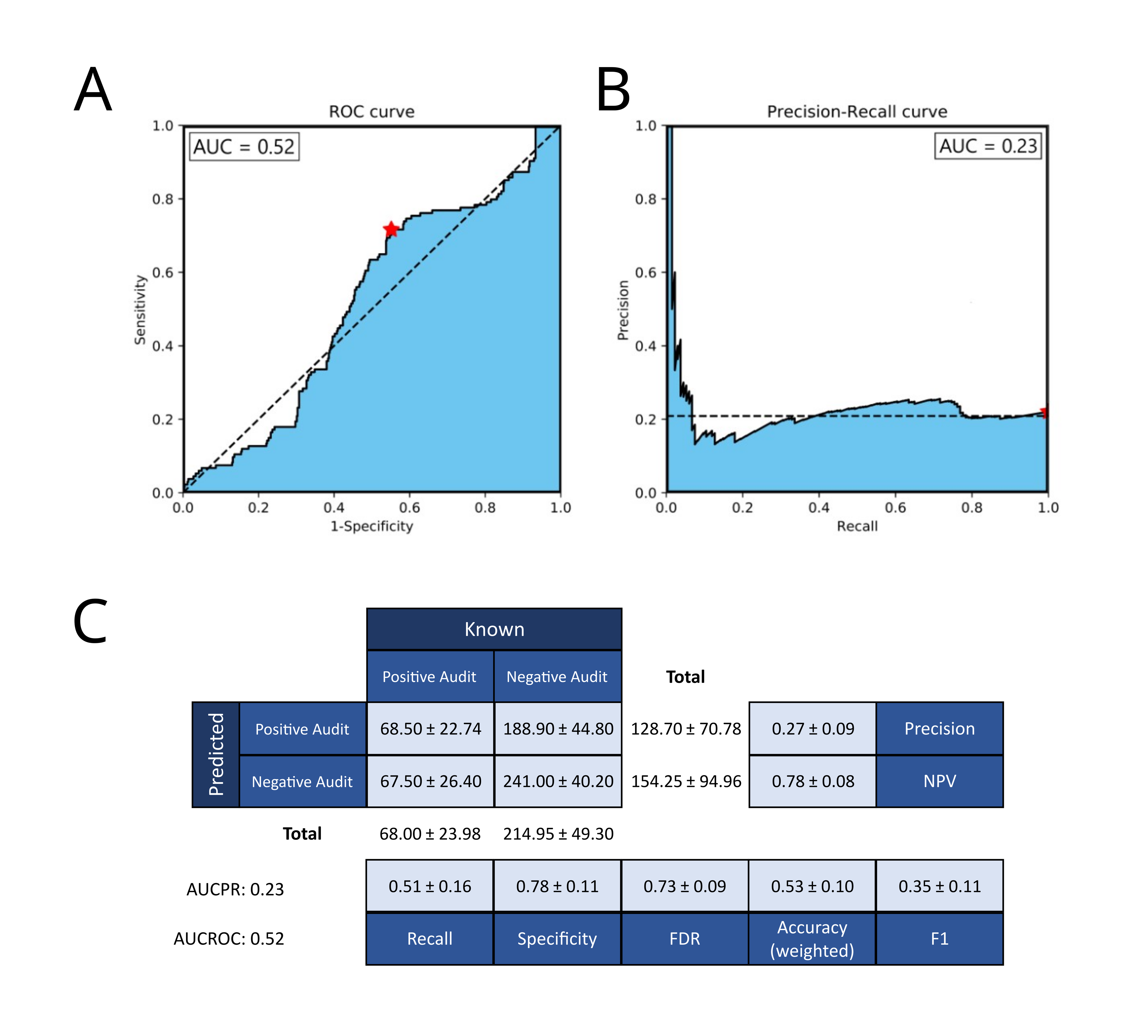

Supplement: S15 Fig — (A) Receiver operating characteristic and (B) precision-recall curves for audit lead classifier without filtration of returns outside of the 95% confidence interval with (C) confusion matrix for classifier trained without filtration of returns outside of the 95% confidence interval. (TIF) [file pone.0278121.s015.tif]

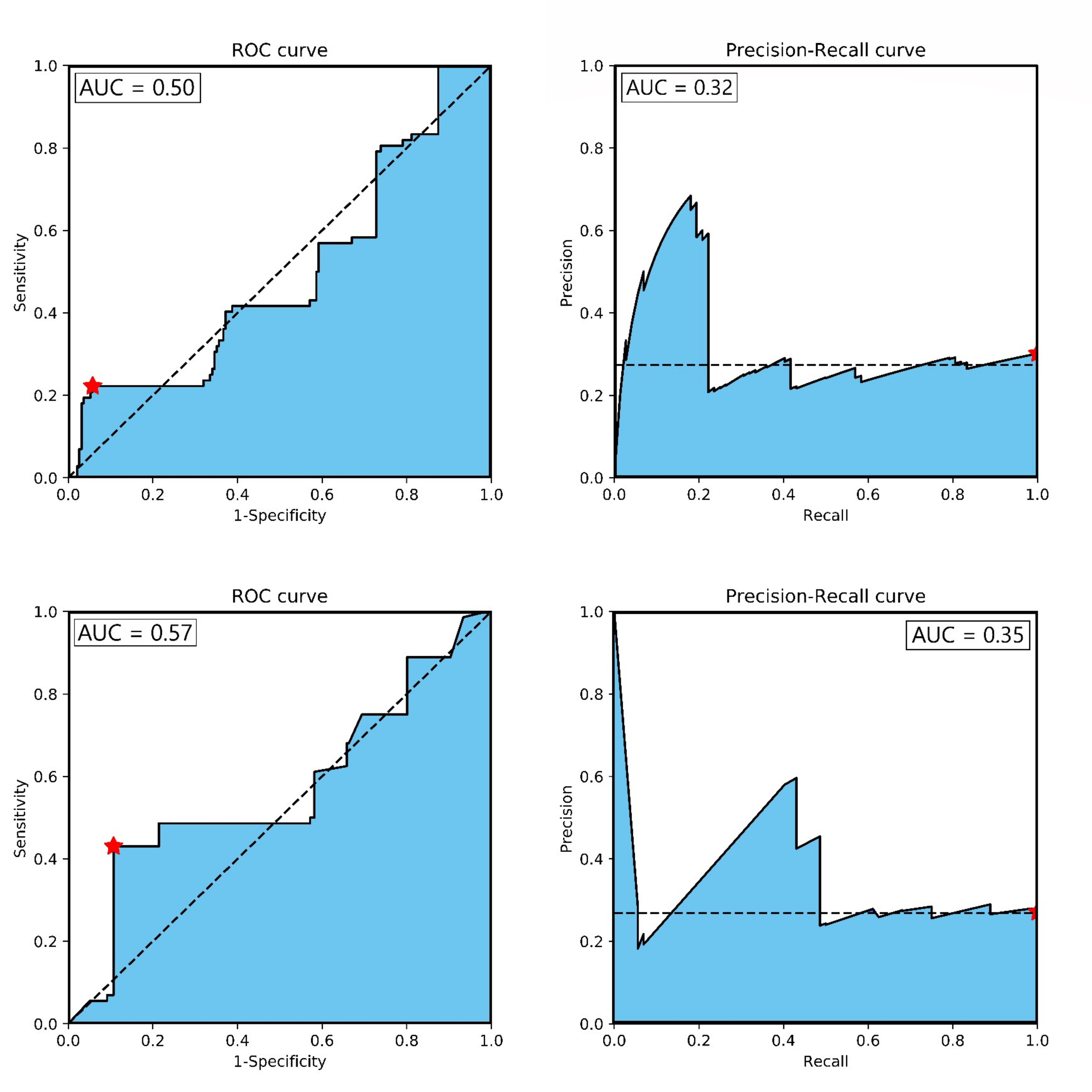

Supplement: S16 Fig — Receiver operating characteristic (ROC) and precision-recall (PR) curves for classifier trained with z-score normalized data (top) and ROC and PR curves for classifier trained with quantile-normalized data (bottom). (TIF) [file pone.0278121.s016.tif]

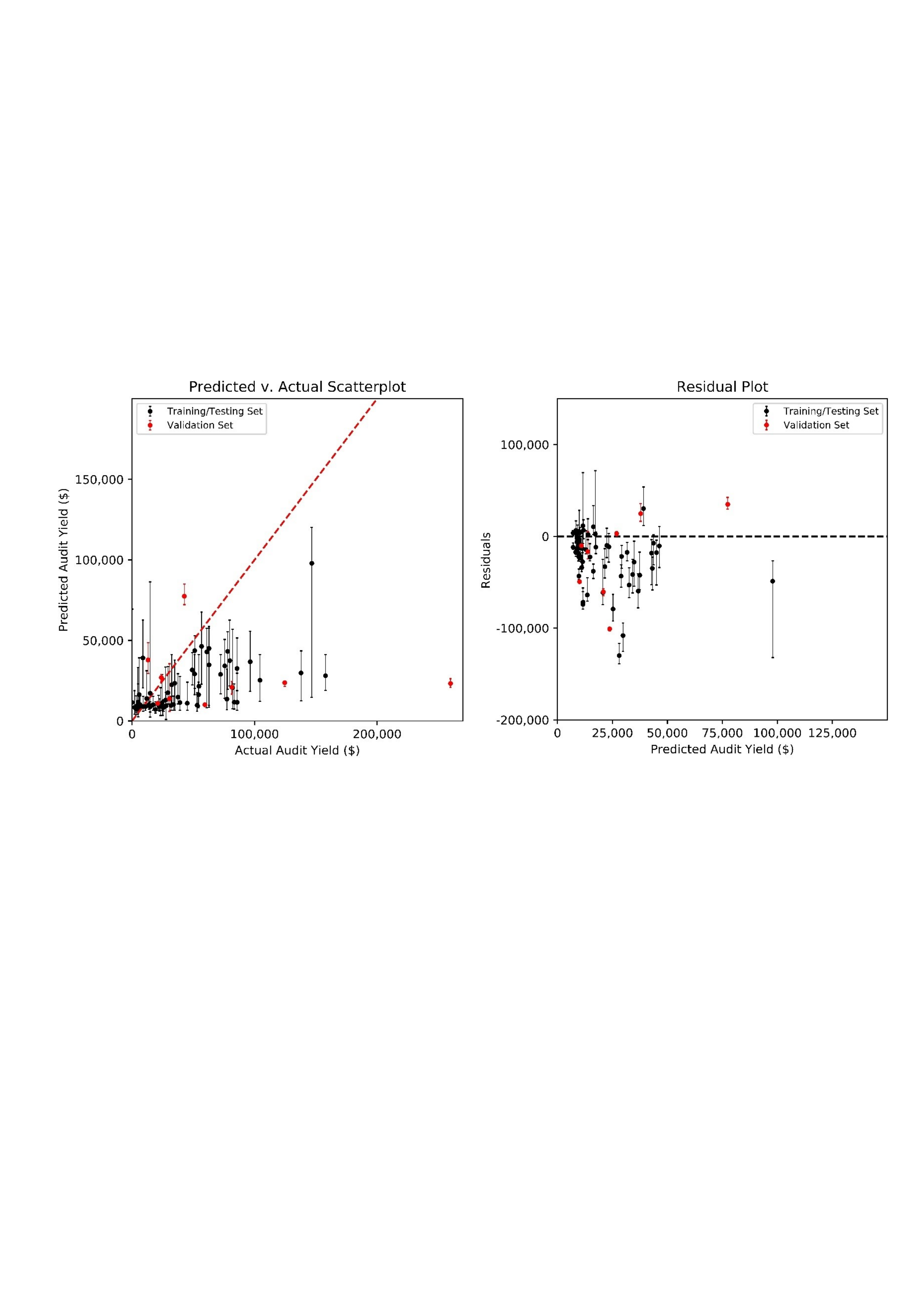

Supplement: S17 Fig — Predicted v. Actual (left) and Residual (right) plots for single regressor model. Error bars represent the prediction on multiple returns, where each point represents the mean of predictions for a single business. (TIF) [file pone.0278121.s017.tif]

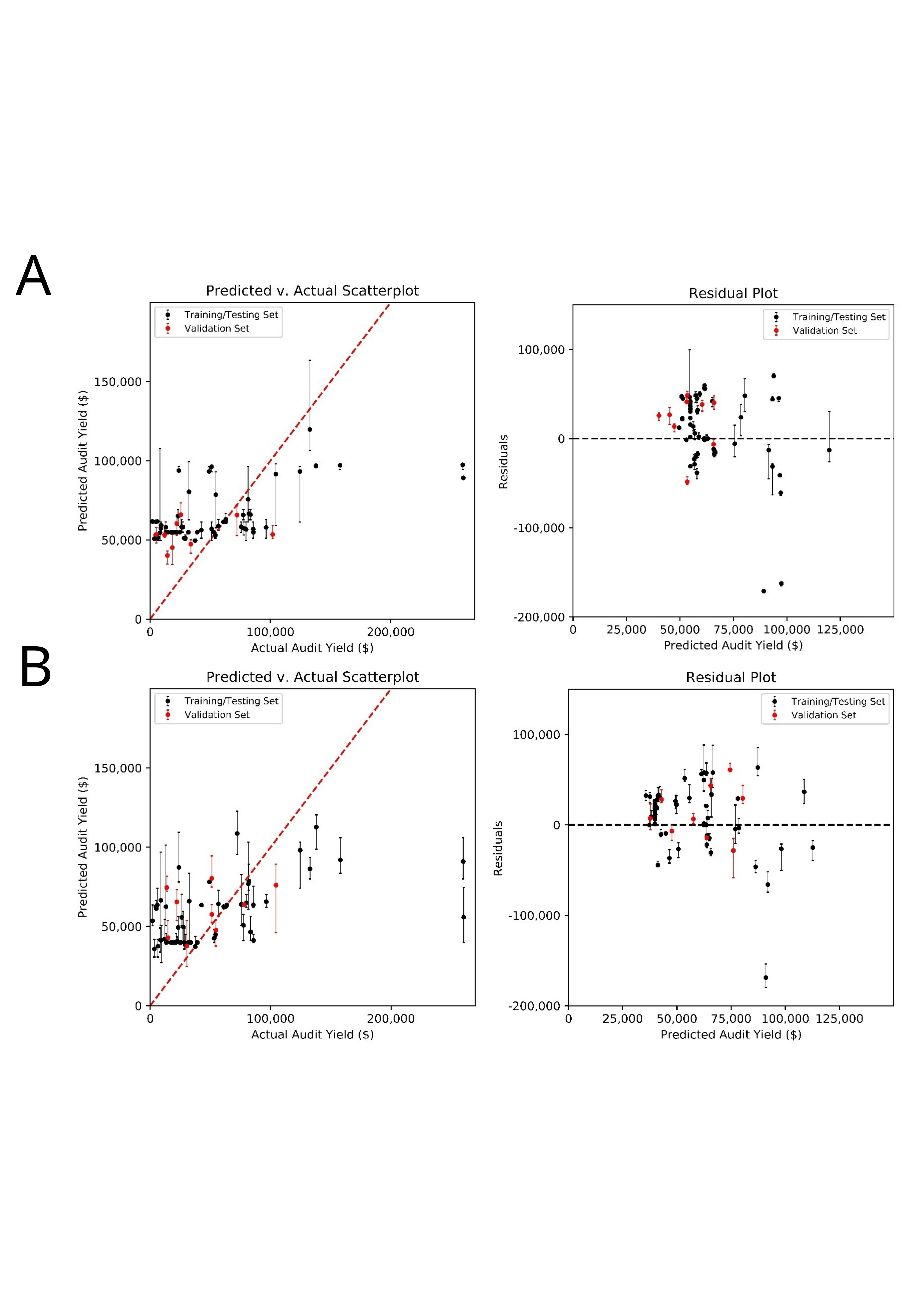

Supplement: S18 Fig — Predicted v. Actual (left) and Residual (right) plots ensemble model created with (A) Hypothesis 1 splits, and (B) Hypothesis 5 splits. Error bars represent the prediction on multiple returns, where each point represents the mean of predictions for a single business. (TIF) [file pone.0278121.s018.tif]

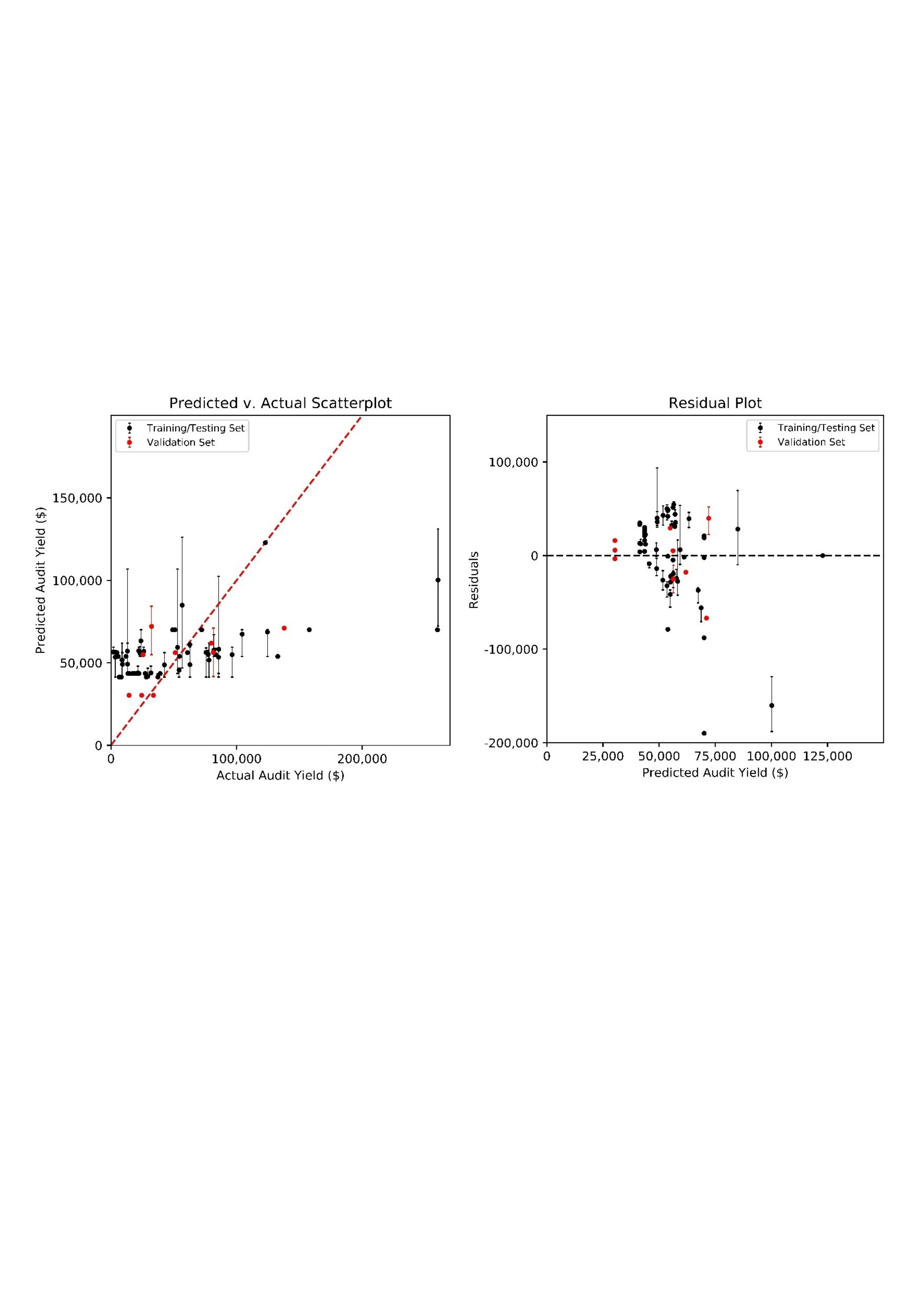

Supplement: S19 Fig — Predicted v. Actual (left) and Residual (right) plots ensemble model created with splits on business type. Error bars represent the prediction on multiple returns, where each point represents the mean of predictions for a single business. (TIF) [file pone.0278121.s019.tif]
