# Supplementary material for: Audit lead selection and yield prediction from historical tax data using artificial neural networks
Source: PLoS One. 2022 Nov 30;17(11):e0278121. doi: 10.1371/journal.pone.0278121 (PMC9710839; doi:10.1371/journal.pone.0278121)
Supplement: S2 File — (DOCX) [file pone.0278121.s021.docx]

Audit lead selection and yield prediction from historical tax data

Trevor Chan^1,2^, Cheng-En Tan^1,2^, and Ilias Tagkopoulos^1,2*^

^1^Department of Computer Science, University of California, Davis 95616, USA

^2^Genome Center, University of California, Davis 95616, USA

*Corresponding author (itagkopoulos@ucdavis.edu)

Supplementary Materials

Table of Contents

[1. HYPOTHESIS TESTING 17](#_Toc42844242)

[2. TAX PREDICTION PIPELINE 17](#_Toc42844243)

[2.1 Overview 17](#_Toc42844244)

[2.2 Dataset 18](#_Toc42844245)

[2.2.1 Filtration 18](#_Toc42844246)

[2.2.2 Visualization 18](#_Toc42844247)

[2.3 Classification Model 19](#_Toc42844248)

[2.3.1 Incorporating All Features 19](#_Toc42844249)

[2.3.2 Without Filtration 20](#_Toc42844250)

[2.3.3 Normalization Methods 20](#_Toc42844251)

[2.4 Regression Model 20](#_Toc42844252)

[2.4.1 Single Regressor Model 20](#_Toc42844253)

[2.4.2 Ensemble Regressor Model 21](#_Toc42844254)

# **1. HYPOTHESIS TESTING**

We examined the following five hypotheses created by the California Department of Tax and Fee Administration’s (CDTFA) auditors:

1. Businesses with gross sales lower than a threshold (calculated as the average in the area based on NAICS code, zip ID, city ID) have higher audit yield than businesses with gross sales higher than said threshold
2. Individual taxpayers are more likely to have positive audits compared with corporations
3. Businesses with the taxable sales/gross sales ratio less than a threshold have higher audit yield than businesses with a said ratio higher than said threshold
4. Variance of reported gross sales (in a specific period) lower than some threshold have higher audit yield than the variance of reported gross sales higher than said threshold
5. Late return penalties that are higher than a threshold have higher audit yield than late return penalties lower than said threshold

As our dataset consists of multiple tax returns per business (every entry for a given audit period had the same audit yield), to prevent overrepresentation of businesses with more return information, data was summed and normalized by the number of returns per audit period. Tables S2, S3, 1 and 2 show statistical validation of the hypotheses and their inverses. We note that while we cannot draw conclusions about hypothesis 3, certain threshold values of the taxable sales, gross sales ratio can be statistically shown to exhibit higher audit yields (Table S4).

# **2. TAX PREDICTION PIPELINE**

## **2.1 Overview**

Provided by a cohesive audit dataset by the California Department of Tax and Fee Administration (CDTFA), we have created a pipeline to generate a comprehensive compendium and predict tax audit yield for a given business. This occurs in several steps (Fig 1). First the data is preprocessed via refinement criteria, normalization and outlier filtration. Features are also filtered based on correlation with other features. Second, the preprocessed dataset is fed as input to a classifier trained on data with audit information to identify which audit leads may or may not be positive. Lastly, audits classified as positive are fed as input to a regressor trained on data with positive audit yield, and audit yield predictions are generated.

## **2.2 Dataset**

The California Department of Tax and Fee Administration provided us with a dataset of 108,162 unique tax records and 42 features. A more concise description of these features can be found in Supplementary File, Data Description Sheet, Table 1 and S1 Fig. Of these 42 features, only 25 were used in the final dataset, as it was decided by the CDTFA that this study would only concern single-location businesses. Additionally, datetime data was not particularly beneficial as we did not use a time-series approach to analyze data (Table S1) We hope to extend our study to accommodate these features in the future.

### **2.2.1 Filtration**

Both feature and outlier filtration were employed. Regarding feature filtration, Pearson correlation was used to measure the level of correlation between features. Features that exhibited a high correlation (Pearson correlation greater than 0.95) were removed, as they do not contribute more to the data than their correlated counterparts [1] (S2 Fig). Outlier filtration was done by first visualizing the distribution of each numerical feature (S3 Fig), and then removing data above the 95% confidence interval for each feature. The purpose of data distribution visualization was to remove extreme data points that could detriment analysis. For example, Total Gross Sales values range from $0 to $20,000,000, with most of the data points falling into the range of $0 to $5,000,000. Incorporating points outside of this range gives an inaccurate interpretation of the data and can produce predictive errors in analysis. S4 Fig shows the same distribution graphs after these data points have been removed.

### **2.2.2 Visualization**

Data was first hierarchically clustered and plotted using Principal Component Analysis (PCA) and t-Distributed Stochastic Neighbor Embedding (t-SNE). First all returns were used, however this led to poor visualization and clustering results (S8 Fig). Merging returns by audit period (S9 Fig), as well as outlier removal (Fig 2, S10 Fig) improved clustering and visualization results. Based on the generated clusters, association rule mining via the Apriori algorithm [2] was employed to extract rules for each cluster. Supplementary File, Clustering Data Sheet, Table 2 shows the number of rules extracted per cluster, while Supplementary File, Clustering Data Sheet, Table 3 lists the top ten rules per cluster. As both PCA and t-SNE plots had difficulties exhibiting the characteristics of tax records with positive audit yield, feature-wise quantile normalization was applied to the dataset, orderings of the features were evaluated. Some clusters based on these orders can be observed in the t-SNE plot (S7B Fig). Each cluster represents a specific order of the 20 numeric features. In S7C Fig, each cluster in the t-SNE plot can be interpreted as the order of 20 numerical features in returns. For example, the most majority patterns (the red cluster in S7C Fig) contain 6,894 returns with the ordering of 20 numerical features are as follows:

$$TotalSales=GrossSales=TaxableTransactions >TaxDue=TotalAmountDue=RemainingTax>DistrictTax>NonTaxFoodProd=\left( Other 12 features \right)=0$$

This means that in these 6,894 returns, all sales are taxable. In addition, there are no deductions, interest, or late penalty in these returns.

## **2.3 Classification Model**

All classification models were evaluated using ten-fold cross-validation on a dataset split 90%-10% between training/testing and validation sets. The Adam optimizer was used, with binary cross-entropy as the loss function [3]. L_2_ regularization was used to reduce overfitting. For early stopping, a delta of 0.0001 and patience, the number of epochs with no improvement after which training is stopped, of 10 was used [4]. Due to high class-imbalance, a combination of over-sampling and under-sampling was performed on each fold [5]. Random search was employed to find optimal hyperparameters on a feed-forward artificial neural network with an input layer where the number of input nodes was equivalent to the number of input features, 2 hidden layers, and one output layer with one output node. The sigmoid function was used as an activation for the nodes in each layer.

### **2.3.1 Incorporating All Features**

We first attempted to use all data features, this included all 143 city and zip code features. Of the 6,301 tax records, 3,688 records above the 95% confidence interval were removed and 38 features were removed with 0 standard deviation, giving a dataset of 2,351 tax records for 510 unique businesses. Parameter tuning with random search yielded 53 and 14 hidden nodes per hidden layer, as well as a batch size of 32 and a dropout rate of 40% were used. Training this architecture with the proposed dataset led to overfitting and poor results on the validation set (S14 Fig). Model training and testing accuracy over all 10 folds was 97% with a standard deviation of 4%, and 63% with a standard deviation of 12%, respectively. The training and testing F_1_ scores were 0.67 and 0.28, while validation accuracy was 49% with an F_1_ score of 0.20.

### **2.3.2 Without Filtration**

We also experimented with a non-filtered approach on the dataset. All 6,301 tax records were used, along with all 37 features for 510 unique businesses. Parameter tuning with random search yielded 23 and 11 hidden nodes per hidden layer, and a batch size of 64 with a dropout rate of 30%. A model trained on this dataset yielded training and testing accuracies of 55% with a standard deviation of 5 % and 53% with a standard deviation of 10%, respectively. The training and testing F_1_ scores were 0.38 and 0.35, while validation accuracy was 54% with an F_1_ score of 0.21. S15 Fig shows a significant decrease in performance when compared with a model trained on filtered data (Fig 2).

### **2.3.3 Normalization Methods**

Besides minmax, quantile and z-score normalization were also tested on the data. PCA and t-SNE were both conducted on the normalized data to better visualize the effects of normalization (S16 Fig). Normalization methods were evaluated based on their effects on classifier performance. Tuned hyperparameters for normalizations can be found in Table S6.

## **2.4 Regression Model**

### **2.4.1 Single Regressor Model**

Like the audit lead classifier, a simple feed-forward artificial neural network was initially constructed for audit yield regression. It was evaluated using ten-fold cross-validation on a dataset split 90%-10% between training/testing and validation sets. The Adam optimizer was used with mean squared error as the loss function [2]. L_2_-regularization was used to reduce overfitting. For early stopping, a delta of 0.0001 and patience, the number of epochs with no improvement after which training is stopped, of 10 was used [3]. Random search was employed to find optimal hyperparameters on a feed-forward artificial neural network with a 30-node input layer, 2 hidden layers, and one output layer with one output node. The Rectified Linear Unit (ReLU) function was used as an activation for the nodes in each layer, with the exception of the linear function which was used as an activation for the output node. Training and testing R-squared values were 0.10 and 0.05, respectively, while validation R-squared was 0.02. Due to data limitation, this model performed poorly (S17 Fig) and our approach needed to be adapted.

### **2.4.2 Ensemble Regressor Model**

Data was split based on validated hypothesis rules and categorical variables to create four distinct ensemble models. For validated hypotheses 1 and 5, the gross sales and late penalty hypotheses, data was split above and below the mean of gross sales and late penalty values. Splits for categorical variables were done on business type and NAICS codes, and not on categorical variables such as city ID or zip code as there were too many variables with too few tax records. For splits based on business type, data was split between individual, corporation, and the remaining business entities. More splits were not done due to lack of data – there were 675 corporation tax records, 437 individual tax records, and 382 other tax records. Finally, for splits based on NAICS codes data was split between limited service restaurants, mobile food services, and all other NAICS codes. More splits were not done due to the lack of data – there were 550 limited-service tax records, 241 mobile-service tax records, and 207 remaining NAICS code records. The ensemble model based on NAICS codes performed the best, with a validation R-squared values of 0.34, while the ensemble model generated based on business type performed equally as well, with a validation R-squared of 0.30. The ensemble models based on hypotheses 1 and 5 yielded validation R-squared values of 0.09 and 0.16, respectively. Values of tuned hyperparameters were done by random search (Table S7).

**REFERENCES**

1. Biesiada J., Duch W. (2007) Feature Selection for High-Dimensional Data — A Pearson

Redundancy Based Filter. In: Kurzynski M., Puchala E., Wozniak M., Zolnierek A.

(eds) Computer Recognition Systems 2. Advances in Soft Computing, vol 45. Springer, Berlin, Heidelberg

2. R. Agrawal and R. Srikant, "Fast algorithms for mining association rules", Proc. of the VLDB

Conference

3. Buja, A., Stuetzle, W. and Shen, Y. Loss functions for binary class probability estimation and

classification: structure and applications. manuscript, available at

www-stat.wharton.upenn.edu/~buja/

4. Lutz Prechelt. Early stopping-but when? Neural Networks: Tricks of the trade, pp. 553–553,

1998.

5. Chawla, N. et al. “SMOTE: Synthetic Minority Over-sampling Technique.” J. Artif.

Intell. Res. 16 (2002): 321-357.

| **Index** | **Data Field** | **Reason not Used** |
| --- | --- | --- |
| 1 | Site ID | Only analyzing single location businesses |
| 2 | Business Establishment Date | Only active accounts in this dataset, not a useful piece of data |
| 3 | Business Close Date | Only active accounts in this dataset |
| 4 | Business Establish Start Date (location specific) | Only analyzing single location businesses |
| 5 | Business Close Date (location specific) | Only analyzing single location businesses |
| 6 | Anonymized Selling Location City Name ID | Only analyzing single location businesses |
| 7 | Anonymized Selling Location Zip Code ID | Only analyzing single location businesses |
| 8 | Return Filing Period | Could not find a way to use this data |
| 9 | Return Period Begin | Could not find a way to use this data |
| 10 | Return Period End | Could not find a way to use this data |
| 11 | Return Received Date | Could not find a way to use this data |
| 12 | Return Due Date | Could not find a way to use this data |
| 13 | Return Type | Only one possible entry, removed for redundancy |
| 14 | Form Type | Only analyzing single location businesses |
| 15 | Total nontaxable deductions | Linear combination of features 24-30 |
| 16 | Taxable Sales | Linear combination of features 23-30 |
| 17 | Total Prepayments | Linear combination of features 36-37 |

Table S1: List of 17 features not used, with a brief explanation as to why.

**SUPPLEMENTARY TABLES**

| q-value (0.05 threshold) | Threshold selected (percentage of the mean) | | | | | | |
| --- | --- | --- | --- | --- | --- | --- | --- |
|  | 0.25 | 0.5 | 0.75 | Mean | 1.25 | 1.5 | 1.75 |
| Reported gross sales lower than some threshold result in higher yield audits (vs. when reported gross sales is higher than threshold) | < 10^-2^* | < 10^-4^* | < 10^-4^* | < 0.01* | 0.02* | 0.02* | 0.03* |
| Individual taxpayers are more likely to have positive audits compared to corporations | 0.17 | | | | | | |
| The ratio of taxable to gross sales $\frac{\mathrm{TaxableSales}}{\mathrm{GrossSales}}$is lower than some threshold result in higher yield audits | 0.96 | 0.94 | 0.98 | 0.58 | 0.07 | 0.05 | 0.10 |
| Variance of reported gross sales (in a specific period) lower than threshold result in higher yield audits | 0.1 | 0.40 | 0.31 | 0.11 | 0.34 | 0.27 | 0.17 |
| Late return penalties higher than some threshold result in higher yield audits | < 10^-7^* | < 10^-7^* | < 10^-6^* | < 10^-6^* | < 10^-7^* | < 10^-5^* | < 10^-4^* |

Table S2—Tax audit empirical hypothesis validation

Table S2: Wilcoxon-ranksum test applied to the original hypotheses. A result less than 0.05 (marked by *) signifies a statistically significant result.

Table S3—Tax audit empirical inverse hypothesis validation

| p-value (0.05 threshold) | Threshold selected (percentage of the mean) | | | | | | |
| --- | --- | --- | --- | --- | --- | --- | --- |
|  | 0.25 | 0.5 | 0.75 | Mean | 1.25 | 1.5 | 1.75 |
| Reported gross sales lower than some threshold result in higher yield audits (vs. when reported gross sales is higher than threshold) | 0.99 | 0.99 | 0.99 | 0.98 | 0.98 | 0.98 | 0.97 |
| Individual taxpayers are more likely to have positive audits compared to corporations | 0.84 | | | | | | |
| The ratio of taxable to gross sales$\frac{\mathrm{TaxableSales}}{\mathrm{GrossSales}}$ is lower than some threshold result in higher yield audits | 0.04* | 0.06 | 0.02* | 0.42 | 0.93 | 0.95 | 0.91 |
| Variance of reported gross sales (in a specific period) lower than threshold result in higher yield audits | 0.1 | 0.40 | 0.31 | 0.11 | 0.34 | 0.27 | 0.17 |
| Late return penalties higher than some threshold result in higher yield audits | 0.99 | 0.99 | 0.99 | 0.99 | 0.99 | 0.99 | 0.99 |

Table S3: Wilcoxon-ranksum test applied to the inverse hypotheses. A result less than 0.05 (marked by *) signifies a statistically significant result.

| $R=\frac{Taxable}{GrossSales}$ | # Returns  (Audit Yield = CDTFA Threshold) | # Returns  (Audit Yield > CDTFA Threshold) | Ratio  (Audit Yield > CDTFA Threshold) | p-value  (HyperGeometric) | p-value  (Wilcoxon-ranksum) |
| --- | --- | --- | --- | --- | --- |
| 0.50 $\leq$ $R$ < 0.55 | 80 | 5 | 5.9% | > 1 | > 1-10^-1^ |
| 0.55 $\leq$ $R$ < 0.60 | 39 | 10 | 20.4% | > 1-10^-1^ | > 1-10^-1^ |
| 0.60 $\leq$ $R$ < 0.65 | 43 | 6 | 12.2% | > 1-10^-1^ | > 1-10^-1^ |
| 0.65 $\leq$ $R$ < 0.70 | 50 | 40 | 44.4% | < 10^-6^ | < 10^-6^ |
| 0.70 $\leq$ $R$ < 0.75 | 28 | 12 | 30.0% | > 1-10^-1^ | > 1-10^-1^ |
| 0.75 $\leq$ $R$ < 0.80 | 47 | 32 | 40.5% | < 10^-4^ | < 10^-6^ |
| 0.80 $\leq$ $R$ < 0.85 | 79 | 28 | 26.2% | > 1-10^-1^ | > 1-10^-1^ |
| 0.85 $\leq$ $R$ < 0.90 | 83 | 22 | 21.0% | > 1-10^-1^ | > 1-10^-1^ |
| 0.90 $\leq$ $R$ < 0.95 | 1,463 | 486 | 24.9% | > 1-10^-2^ | > 1-10^-4^ |
| 0.95 $\leq$ $R$ < 1.00 | 276 | 8 | 2.8% | > 1 | > 1 |
| $R$ $\geq$ 1 | 2,093 | 784 | 27.3% | < 10^-10^ | < 10^-2^ |

Table S4: Taxable transaction ratios and their corresponding audit yields. We observe that the ratio of taxable transaction to gross sales in three specific ranges produce higher audit yields. Even for the returns where all gross sales are taxable, about 27.3% of them produce audit yields > CDTFA Threshold (significantly higher than the average, 23.7%)

|  | **Features** | **Used in Classification Model** | **Used in Regression Model** |
| --- | --- | --- | --- |
| 1 | Late Interest | Yes | Yes |
| 2 | Late Penalty | Yes | Yes |
| 3 | Total Gross Sales | Yes | Yes |
| 4 | Total Amount Due | Yes | Yes |
| 5 | Total Sales | Yes | Yes |
| 6 | Nontaxable Sales Tax included in Gross Sales | Yes | Yes |
| 7 | Tax Due | Yes | Yes |
| 8 | Excess Tax | Yes | No |
| 9 | Remaining Tax | Yes | Yes |
| 10 | Use Tax Purchases | Yes | Yes |
| 11 | Sale of Nontaxable Food | Yes | Yes |
| 12 | Monthly 2nd prepayment made | Yes | Yes |
| 13 | Filing Frequency - SUTFA | Yes | No |
| 14 | Filing Frequency - SUTQ | Yes | Yes |
| 15 | Filing Frequency - SUTPQ | Yes | Yes |
| 16 | Filing Frequency - SUTM | Yes | Yes |
| 17 | Business Type - LIMITD | Yes | No |
| 18 | Business Type - IND | Yes | Yes |
| 19 | Business Type - PART | Yes | Yes |
| 20 | Business Type - LLC | Yes | Yes |
| 21 | Business Type - CORP | Yes | Yes |
| 22 | NAICS Code - 722400 | Yes | Yes |
| 23 | NAICS Code - 722330 | Yes | Yes |
| 24 | NAICS Code - 722514 | Yes | No |
| 25 | NAICS Code - 722300 | Yes | No |
| 26 | NAICS Code - 722320 | Yes | Yes |
| 27 | NAICS Code - 722513 | Yes | Yes |
| 28 | NAICS Code - 722310 | Yes | Yes |
| 29 | NAICS Code - 722511 | Yes | Yes |
| 30 | NAICS Code - 722515 | Yes | Yes |
| 31 | NAICS Code - 722410 | No | Yes |

Table S5: Final dataset features used in training dataset for classification (left column) and regression (right column) models.

| **Normalization**  **Method** | **Tuned Hyperparameters** | | | | **Evaluation Metrics** | |
| --- | --- | --- | --- | --- | --- | --- |
|  | **Batch**  **Size** | **Hidden Layer 1**  **(# nodes)** | **Hidden Layer 2**  **(# nodes)** | **Dropout** | **Accuracy** | **F_1_** |
| Z-score | 32 | 24 | 11 | 0.3 | 53% | 0.31 |
| Quantile | 64 | 26 | 7 | 0.2 | 55% | 0.34 |
| Minmax | 32 | 19 | 8 | 0.3 | 61% | 0.42 |

Table S6: Hyperparameters used for models for different normalization methods, along with evaluation results by which models were selected.

Table S7: Hyperparameters used for different ensemble regressors, along with evaluation results by which models were selected.

| **Ensemble Model** | **Tuned Hyperparameters** | | | | | | **Evaluation Metrics** | | |
| --- | --- | --- | --- | --- | --- | --- | --- | --- | --- |
|  | **Features per Tree (%)** | **Learning**  **Rate** | **Samples per Tree (%)** | **Max depth** | **L_1_-regularization value** | **Number of Trees** | **Training R²** | **Testing R²** | **Validation R²** |
| Hypothesis 1 Model | 20 | 0.9 | 30 | 3 | 5 | 30 | 0.22 | 0.13 | 0.09 |
| Hypothesis 5 Model | 20 | 0.8 | 30 | 2 | 5 | 35 | 0.17 | 0.10 | 0.16 |
| NAICS code model | 40 | 1 | 30 | 2 | 5 | 45 | 0.34 | 0.32 | 0.34 |
| Business Type model | 40 | 0.9 | 30 | 2 | 5 | 35 | 0.33 | 0.29 | 0.30 |
